# Associations between cerebrospinal fluid markers and cognition in ageing and dementia: A systematic review

**DOI:** 10.1111/ejn.15656

**Published:** 2022-05-12

**Authors:** Tyler S. Saunders, Danni A. Gadd, Tara L. Spires‐Jones, Declan King, Craig Ritchie, Graciela Muniz‐Terrera

**Affiliations:** ^1^ UK Dementia Research Institute The University of Edinburgh Edinburgh UK; ^2^ Center for Discovery Brain Sciences The University of Edinburgh Edinburgh UK; ^3^ Center for Clinical Brain Sciences The University of Edinburgh Edinburgh UK; ^4^ Center for Dementia Prevention The University of Edinburgh Edinburgh UK; ^5^ Center for Genomic and Experimental Medicine, Institute of Genetics and Molecular Medicine University of Edinburgh Edinburgh UK

**Keywords:** Alzheimer disease, biomarkers, cerebrospinal fluid, cognition, cognitive aging, dementia

## Abstract

A biomarker associated with cognition in neurodegenerative dementias would aid in the early detection of disease progression, complement clinical staging and act as a surrogate endpoint in clinical trials. The current systematic review evaluates the association between cerebrospinal fluid protein markers of synapse loss and neuronal injury and cognition. We performed a systematic search which revealed 67 studies reporting an association between cerebrospinal fluid markers of interest and neuropsychological performance. Despite the substantial heterogeneity between studies, we found some evidence for an association between neurofilament‐light and worse cognition in Alzheimer's diseases, frontotemporal dementia and typical cognitive ageing. Moreover, there was an association between cerebrospinal fluid neurogranin and cognition in those with an Alzheimer's‐like cerebrospinal fluid biomarker profile. Some evidence was found for cerebrospinal fluid neuronal pentraxin‐2 as a correlate of cognition across dementia syndromes. Due to the substantial heterogeneity of the field, no firm conclusions can be drawn from this review. Future research should focus on improving standardization and reporting as well as establishing the importance of novel markers such as neuronal pentraxin‐2 and whether such markers can predict longitudinal cognitive decline.

## Graphical abstract

We conducted a systematic review of studies examining the association between cerebrospinal fluid markers and neuropsychological performance. Despite substantial heterogeneity in the field, there was some evidence of an association between cognition and neurofilament‐light and neurogranin. Risk of bias in the field requires improvement (Created with Biorender.com).

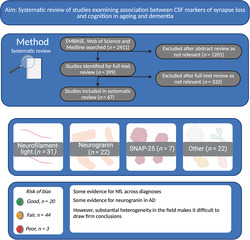

## 1 INTRODUCTION

Dementia is a syndrome characterised by progressive cognitive decline. An estimated 50 million people are living with a form of dementia worldwide, which is expected to reach 82 million by 2030 (World Health Organisation, 2020). The identification of a biomarker which correlates with cognition would have numerous benefits. An earlier indication of the pathophysiological processes underlying cognitive impairment is needed, as neuronal loss precedes detectable cognitive symptoms and so may be used to predict prognosis (Counts et al., 2017; DeKosky & Marek, 2003). Moreover, such markers could benefit our aetiological understanding of dementias as different synaptic markers could reflect different pathophysiological mechanisms. Next, in clinical trials, they could be used as surrogate endpoints for synapse‐targeting pharmacological interventions and could aid in the selection of participants who are in the earliest stages of dementia (Atri, 2011; Yiannopoulou & Papageorgiou, 2013). However, at present, there are no widely used biomarkers that predict cognitive status or cognitive decline in dementias.

Alzheimer's disease, the leading cause of dementia (World Health Organisation, 2020), is characterised by the pathological hallmarks of extracellular deposition of amyloid‐β (Aβ), intracellular accumulation of abnormally hyperphosphorylated tau into neurofibrillary tangles and brain atrophy due to neuronal and synapse loss (Blennow et al., 2006). These hallmarks of AD are present in mild cognitive impairment (MCI) and even before detectable symptoms begin to emerge—with Aβ accumulation possibly beginning up to two decades before symptom manifestation (Counts et al., 2017; Jack et al., 2010). Changes in the levels of these pathological proteins in the cerebrospinal fluid (CSF) have been observed as they aggregate in the brain and so the CSF may be a viable source of potential biomarkers.

The cerebrospinal fluid is a clear liquid which surrounds the brain and provides mechanical support, transfers micronutrients and signalling molecules to neurons and is involved in the removal of unnecessary metabolites (Spector et al., 2015). The CSF is an ideal source for biomarkers associated with cognition as it directly interacts with the extracellular space of the brain and so it can reflect the occurrence of pathophysiological changes (Hampel et al., 2012). In AD, the deposition of extracellular Aβ is reflected by reduced CSF levels of the 42‐amino acid form of Aβ (Aβ_42_) or the Aβ_42_/Aβ_40_ ratio, likely reflecting the reduced clearance of the protein (Potter et al., 2013; Tarasoff‐Conway et al., 2015). In contrast, levels of both total tau (t‐tau) and phosphorylated tau (p‐tau) are increased in the brain and in the CSF in AD (Counts et al., 2017; Ortega et al., 2019; Savage et al., 2014). These core CSF biomarkers of AD have high diagnostic accuracy (Counts et al., 2017; Ortega et al., 2019; Savage et al., 2014) and can predict conversion from MCI to AD (Caminiti et al., 2018; Li et al., 2016; Ortega et al., 2019). Indeed, they are currently accepted in international diagnostic criteria for use in the research diagnosis of AD and pre‐clinical AD (Dubois et al., 2014; Jack et al., 2018). However, despite the utility of these core CSF biomarkers as diagnostic tools, they correlate weakly with cognitive impairment. Studies report weak or no significant associations between cognitive performance and CSF Aβ (Kester et al., 2009; Ottoy et al., 2019; Zhou et al., 2009) and moderate‐to‐poor relationships with CSF t‐tau and p‐tau (Buchhave et al., 2009; Ecay‐Torres et al., 2018; Mattsson, Schöll, et al., 2017; Wattmo et al., 2020; Zhou et al., 2009). Meanwhile, other neurodegenerative dementias such as frontotemporal dementia (FTD), vascular dementia (VaD) and dementia with Lewy bodies (DLB) also lack a validated biomarker that associated with cognition. For example, CSF t‐tau and p‐tau can accurately discriminate FTD from controls (Meeter, Vijverberg, et al., 2018) but only have a moderate‐to‐weak correlation with neuropsychological performance (Bian et al., 2008; Borroni et al., 2011; Goossens et al., 2018). Accordingly, there is a need for additional validated CSF biomarkers which correlate with cognition and biomarkers of synapse loss that have been proposed as potential candidates.

Healthy synapse function enables neuronal signal transmission to occur, which is facilitated by pre‐synaptic and post‐synaptic compartments. Synaptic plasticity, formation, maturation and elimination involve processes essential for learning and memory, namely, long‐term potentiation (LTP) and long‐term depression (LTD) (Bear & Malenka, 1994). LTP refers to the strengthening of synaptic transmission by the addition of new receptors at the post‐synaptic density and the enlargement of dendritic spine heads. Conversely, LTD refers to the weakening of synaptic strength and spine shrinkage/loss (Citri & Malenka, 2008). The total number of synapses in the brain decreases with typical ageing, which is exacerbated in AD and other dementias (Bertoni‐Freddari et al., 1990; DeKosky & Scheff, 1990; Masliah et al., 1994, 2006). What is more, synapse loss is the strongest pathological correlate of cognitive decline in AD (De Wilde et al., 2016; DeKosky & Scheff, 1990; Masliah et al., 1994; Terry et al., 1991). Accordingly, CSF markers of synapse loss would be expected to correlate with cognitive impairment. Indeed, a number of CSF synapse and neuronal marker levels are altered in dementia syndromes and age‐related cognitive decline, some of which will be discussed. *Before continuing, it is important to note that any CSF biomarker associated with cognition is primarily a marker of changes in the brain. Such pathophysiological changes may lead to neuronal network breakdown/damage, which may translate into cognitive symptoms at a point in the future. Therefore, the term ‘biomarker for cognition’ is erroneous and should be avoided*.

### 1.1 Neurofilament‐light

Neurofilaments are classed as type IV intermediate filaments and are primarily located in axons. They play essential roles in radial growth, cytoskeletal support and transmission of electrical impulses along axons (Fuchs & Cleveland, 1998; Petzold, 2005). Neurofilaments are heteropolymers and are composed of four subunits in the CNS: neurofilament‐light (NfL), neurofilament‐medium (NfM), neurofilament‐heavy (NfH) and α‐internexin, of which NfL is the essential component. CSF NfL has been established as a general marker of axonal damage across neurodegenerative diseases as NfL is released into the extracellular fluid following axonal injury (Petzold, 2005). Indeed, CSF NfL levels correlate with brain atrophy (Dhiman et al., 2020; Pereira et al., 2017) and are elevated across dementias, MCI (Olsson et al., 2016; Petzold et al., 2007; Rosengren et al., 1999; Zetterberg et al., 2016) and neurodegenerative diseases such as amyotrophic lateral sclerosis (ALS) and Parkinson's disease (PD) (Gaetani et al., 2019).

### 1.2 Neurogranin (Ng)

Ng is a post‐synaptic peripheral membrane protein involved in LTP and memory formation. Ng binds calmodulin (CaM) in the absence of calcium (Ca^2+^) and thus regulates CaM availability (Petersen & Gerges, 2015). In the AD brain, full‐length Ng levels are reduced (Kvartsberg et al., 2019; Reddy et al., 2005), whereas CSF levels are increased in AD and MCI (Dulewicz et al., 2020). Elevated CSF Ng levels appear to be specific to AD, rather than reflecting general synapse damage in other neurodegenerative diseases or dementias (Portelius et al., 2018; Wellington et al., 2016).

### 1.3 Pre‐synaptic and neuronal markers

Cerebrospinal fluid levels of proteins localised at the pre‐synapse and post‐synapse are an obvious choice for a CSF marker of synapse loss/damage. The localization and normal function of such proteins suggest that they could be adequate surrogate markers for synapse loss, as they may be released into the extracellular fluid following synapse damage (Vergallo et al., 2018). Both NfL and Ng are some of the most researched markers. Next, we briefly discuss other pre‐synaptic and neuronal markers with a short description of their function, localization and potential roles in dementia syndromes.

Alpha‐synuclein (α‐syn) is a pre‐synaptic protein, expressed predominately in the neocortex and subcortical areas, including the hippocampus (Emamzadeh, 2016; Kim et al., 2014). Aggregates of hyperphosphorylated, misfolded α‐syn are the main component of Lewy bodies (LBs), the characteristic pathological accumulates of α‐synucleinopathies such as PD, Parkinson's disease dementia (PDD) and DLB (Kim et al., 2014). The normal function of α‐syn is not fully understood; however, it is thought to be involved in vesicle fusion and neurotransmitter release (Kim et al., 2014). The localization and normal function of α‐syn suggests that it could be used as a surrogate marker for synapse loss as it may be released into the extracellular fluid following synapse damage (Vergallo et al., 2018). Studies measuring full‐length α‐syn (rather than LB‐specific fragments) report significant elevations in AD and MCI and those with α‐synucleinopathies (Hansson et al., 2014; Korff et al., 2013; Slaets et al., 2014).

Beta‐synuclein (β‐syn) is a pre‐synaptic protein which is highly enriched in the hippocampus (Uhlén et al., 2015). It is homologous to and co‐localises with α‐syn (Williams et al., 2018). The normal function of β‐syn is unknown, although there is evidence to suggest that it has a role in the inhibition of α‐syn aggregation (Williams et al., 2018). Independent of its pathological form, β‐syn may be a good marker of synapse loss due to its localization at the pre‐synapse.

Contactin‐2 is a pre‐synaptic and axonal protein (Furley et al., 1990), expressed in frontal and temporal lobes—including hippocampal pyramidal cells (Gautam et al., 2014; Murai et al., 2002). Contactin‐2 is involved in axonal guidance during development, neuronal fasciculation and axonal domain organisation (Masuda, 2017; Wolman et al., 2008). In AD, contactin‐2 levels are reduced in the brain (Chatterjee, Del Campo, et al., 2018; Gautam et al., 2014) and altered in the CSF, although findings are somewhat discrepant with regard to whether CSF levels are elevated or decreased (Chatterjee, Del Campo, et al., 2018; Yin et al., 2009). Contactin‐2 may be a potential marker of general synapse and axonal damage for neurodegenerative diseases as CSF levels are also increased in multiple sclerosis (MS) (Chatterjee, Koel‐Simmelink, et al., 2018).

GAP‐43 is a pre‐synaptic protein widely expressed in the CNS during the development, which reduces with maturation (Holahan, 2017). In adulthood, GAP‐43 is expressed in hippocampal pyramidal cells and association cortices (Chung et al., 2020; Neve et al., 1988; Riascos et al., 2014) and is involved in axonal outgrowth, synaptic plasticity and functions associated with learning and memory (Chung et al., 2020; Holahan, 2017). Levels of GAP‐43 in the frontal cortex are reduced in a number of dementia syndromes (Bogdanovic et al., 2000; Davidsson & Blennow, 1998; Rekart et al., 2004). Moreover, CSF GAP‐43 levels are increased in AD, FTD‐syndromes (Remnestål et al., 2016) and other neurodegenerative diseases such as PD and ALS (Sandelius et al., 2019).

The neuronal pentraxin family includes neuronal pentraxin I (NPTX1), neuronal pentraxin 2 (NPTX2) and neuronal pentraxin receptor (NPTXR) which are highly enriched in excitatory pyramidal neurons of the hippocampus and cerebellum (Chang et al., 2010; Dodds et al., 1997). All three neuronal pentraxins are involved in developmental and adult synaptic plasticity, formation and remodelling, as well as the maintenance of parvalbumin interneuron activity (Chang et al., 2010; Osera et al., 2012). NPTX1/2 are secreted pre‐synaptic proteins, whereas NPTXR is a membrane‐anchored protein (Lee et al., 2017). In the brain and the CSF, NPTX2 levels are reduced in AD, MCI, FTD and aged controls (Soldan et al., 2019; van der Ende et al., 2020, 2019; Xiao et al., 2017).

Neuregulin 1 (nrg1), a substrate of BACE1, is a pre‐synaptic protein thought to be implicated in a number of neurodegenerative diseases and psychiatric/neurodevelopmental disorders such as AD, attention deficit hyperactive disorder (ADHD) and schizophrenia (Shi & Bergson, 2020). Nrg1 is thought to be involved in synaptic transmission and plasticity (Fischbach, 2007); however, at least 31 isoforms have been described which all perform a broad range of functions throughout the body. It is unclear whether Nrg1 in the brain exerts protective or detrimental effects on cognition as both high and low levels of Nrg1 at synapses lead to cognitive impairment in animal models (Agarwal et al., 2014). There are no known human post‐mortem brain studies examining Nrg1 levels in dementias; however, elevations of CSF Nrg1 have been reported in AD and MCI (Mouton‐Liger et al., 2020; Pankonin et al., 2009).

Synaptosomal‐associated protein 25 (SNAP‐25) is a pre‐synaptic protein involved in vesicular exocytosis, LTP and the formation of SNARE complexes (Noor & Zahid, 2017). In post‐mortem brain studies, levels of SNAP‐25 are reduced across dementia syndromes (Connelly et al., 2011; Minger et al., 2001; Mukaetova‐Ladinska et al., 2009; Sinclair et al., 2015). Levels of CSF SNAP‐25 are increased in AD and MCI (Brinkmalm et al., 2014; Galasko et al., 2019; Wang, Zhou, & Zhang, 2018; Zhang, Therriault, et al., 2018), potentially reflecting the release of SNAP‐25 from synapses into the extracellular space. Elevations have also been reported in PD, Creutzfeldt‐Jakob Disease (CJD) (Noor & Zahid, 2017) and a number of psychiatric disorders; hence, CSF SNAP‐25 could be a general marker of synapse damage (Najera et al., 2019).

Synaptotagmin‐1 is a pre‐synaptic protein involved in synaptic vesicle exocytosis and synaptic transmission (Baker et al., 2015; Jahn & Fasshauer, 2012). Across dementia syndromes, synaptotagmin‐1 levels are reduced in the brain (Bereczki et al., 2018; Davidsson & Blennow, 1998; Yoo et al., 2001) and elevated in the CSF (Öhrfelt et al., 2016, 2019; Tible et al., 2020).

Visinin‐like protein‐1 (VILIP‐1) is a neuronal calcium sensor protein which is widely expressed in neurons and involved in signalling pathways related to synaptic plasticity (Braunewell, 2012). In AD and FTD, VILIP‐1 expression is reduced in the temporal/entorhinal cortices (Braunewell et al., 2001; Kirkwood et al., 2016) and the superior frontal gyrus, respectively (Kirkwood et al., 2016). Additionally, in the CSF, a recent meta‐analysis reported elevated CSF VILIP‐1 levels in AD and MCI due to AD (Dulewicz et al., 2020).

To date, there is no summary of the evidence examining the relationship between CSF markers of synapse loss and neuronal damage and cognition in ageing and disease. Hence, we conducted a systematic review examining the scientific literature for associations between these markers and cognition in healthy ageing and dementia syndromes. We searched for papers examining any type of dementia or cognition in typical ageing to characterise the cross‐diagnostic specificity of markers. Levels of CSF Aβ or tau were not considered as this was beyond the scope of the current review. We searched for correlates of both cross‐sectional cognition only.

## 2 MATERIALS AND METHODS

The protocol for this review was prospectively registered on PROPSERO (CRD42020164456).

### 2.1 Search strategy

The initial search was conducted in December 2019 within MEDLINE, EMBASE and Web of Science. The most recent update search was conducted on 4 January 2021. Search terms can be found in the supporting information Table S1. Reference lists of studies and reviews were manually searched to identify additional studies. No restrictions were applied for language or date of publication. Only published studies in peer reviewed journals were included; conference abstracts were excluded.

### 2.2 Eligibility criteria

The inclusion criteria were that the study: (i) included a population with a diagnosis of Alzheimer's disease, MCI, FTD, any other type of dementia or a cognitively unimpaired (CU) sample; (ii) measured a cerebrospinal fluid marker of synapse loss and/or neuronal damage, excluding Aβ or tau; (iii) assessed cognition using a validated tool; and (iv) directly examined the relationship between the CSF marker and cognition.

Exclusion criteria included studies (i) where participants were diagnosed with a psychiatric disorder, (ii) review articles, (iii) conference abstracts, (iv) animal studies and (v) studies which only examined CSF Aβ or tau.

Two researchers (T.S.S. and D.A.G.) independently screened studies for inclusion/exclusion and resolved any discrepancies through discussion.

### 2.3 Data extraction

T.S.S. and D.A.G. independently extracted data from eligible studies using Covidence software. This included the following: year of publication, demographics, sample size, medication status, apolipoprotein E (ApoE) status, mean/median CSF marker levels with the appropriate measure of variation and other related information. Researchers were not blinded to authors, journals or institutions. Any discrepancies were resolved by discussion and joint data extraction. Authors were contacted for additional clarification and to request missing data wherever possible.

### 2.4 Risk of bias assessment

The Cochrane network advise against quality scales which generate a summary score and instead suggest placing importance on how each study performed on individual criterion (Boutron et al., 2020). Therefore, we assessed the risk of bias in study design and reporting using the National Institute of Health Quality Assessment Tool for Observational Cohort and Cross‐Sectional Studies (National Institutes of Health, 2014). T.S.S. and D.A.G. independently assessed risk of bias, and any discrepancies were resolved by discussion.

### 2.5 Synthesis of results

Correlation coefficients were selected as the standardised metric of the review. After extraction of results, a meta‐analysis was not conducted due to substantial differences in study methodologies and a lack of reporting of correlation coefficients in published reports. Therefore, we grouped studies according to the CSF marker being measured due to a number of studies pooling participants across diagnostic groups in statistical analysis.

## 3 RESULTS

### 3.1 Search results

**FIGURE 1:**
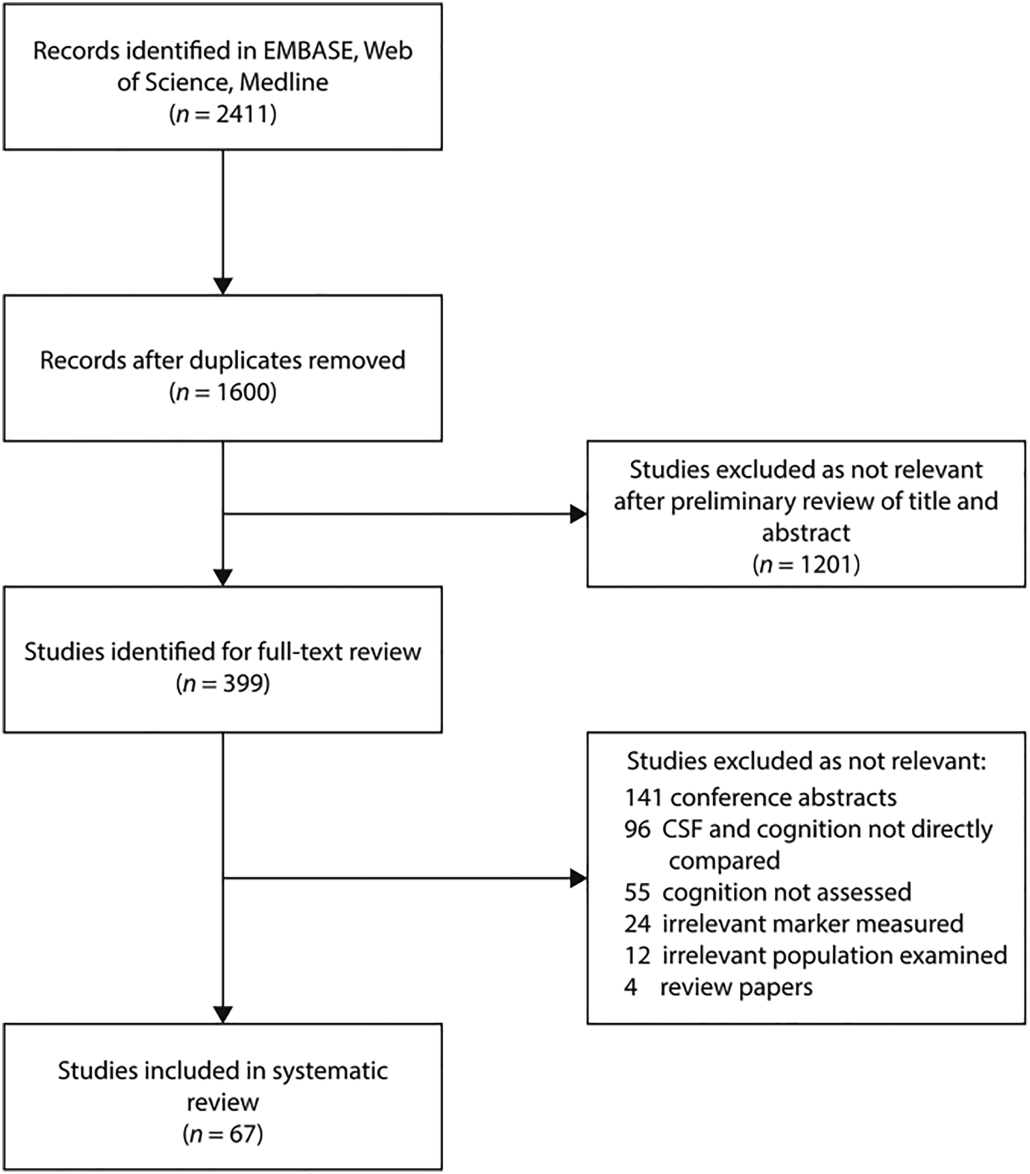
Search process

Two thousand, four hundred and eleven studies were identified. After screening studies for eligibility, 67 studies met criteria for inclusion in the systematic review (see Figure 1).

### 3.2 Study characteristics

#### 3.2.1 Sample size

Characteristics of included studies can be found in Table 1. Some cohorts were used in multiple studies. Ten studies used the Alzheimer's Disease Neuroimaging Initiative (ADNI) (Galasko et al., 2019; Headley et al., 2018; Mattsson et al., 2016; Petersen et al., 2010; Portelius et al., 2015; Sun et al., 2016; Swanson et al., 2016; Wang, 2019; Wang, Zhou, & Zhang, 2018; Zetterberg et al., 2016; Zhang, Ng, et al., 2018; Zhang, Therriault, et al., 2018), five used the Amsterdam Dementia Cohort (Boiten et al., 2021; Chatterjee, Del Campo, et al., 2018; Kvartsberg, Duits, et al., 2015; Meeter, Vijverberg, et al., 2018; van Der Flier & Scheltens, 2018; van Steenoven et al., 2020), three used the Wisconsin Registry for Alzheimer's Prevention (Bendlin et al., 2012; Casaletto et al., 2017; Racine et al., 2016; Sager et al., 2005) and three used the Genetic Frontotemporal Dementia Initiative (GENFI—The Genetic Frontotemporal Initiative) (GENFI – The Genetic Frontotemporal Initiative, n.d.; Meeter et al., 2016; Meeter, Vijverberg, et al., 2018; van der Ende et al., 2020). The Gothenburg Mild Cognitive Impairment Study (Bjerke et al., 2009; Brinkmalm et al., 2014; Rolstad, Berg, et al., 2015; Wallin et al., 2016) was used in three studies, the Mayo Clinic Study of Ageing (Mielke, Syrjanen, Blennow, Zetterberg, Skoog, et al., 2019; Mielke, Syrjanen, Blennow, Zetterberg, Vemuri, et al., 2019; Roberts et al., 2008) in two studies, the Vanderbilt Memory and Ageing Project (Gifford et al., 2018; Jefferson et al., 2016; Osborn et al., 2019) in two studies and finally, the University of California San Diego (UCSD) Shiley‐Marcos Alzheimer's Disease Research Center (Galasko et al., 2019; Xiao et al., 2017) in two studies.

**TABLE 1:** Characteristics of included studies

Study	CSF Marker	CSF analysis assay and brand	Population (N)	Age (years)*	Sex (N, % female)	CSF marker level (pg/mL)*:			Cognitive assessment	Adjustment factors
Abu‐Rumeileh et al., 2018	NfL	ELISA (IBL, Germany)	AD (60) FTD (141)	67.1 (8.7) 64.9 (9.8)	27 (45%) 66 (46.8%)	Median [IQR] 2160 [1614‐2878] 3293 [2120‐7596]			BMDB, FAB	None
Agnello et al., 2020	Ng Alpha‐Synuclein	ELISA (ADx Neurosciences, Belgium)	AD (29)	67.8 (6.4)	15 (51.7%)	Median [IQR]			MMSE	None
						Ng: 460 [410‐647]	α‐syn: 2844[2326.9‐3524.5]			
Alcolea et al., 2017	NfL	ELISA (UmanDiagnostics, Sweden)	FTD (249)	67.12 (8.87)	121 (48.4%)	2069.39 (1833.92)			MMSE	None
Aschenbrenner et al., 2020	NfL	ELISA (UmanDiagnostics, Sweden)	CU Aβ + (94) CU Aβ ‐ (161)	67.31 (8.99) 65.60 (8.47)	40 (43%) 100 (62%)	1356.29 (574.42) 1505.72 (703.91)			Global, episodic memory, attention composites	Age, amyloid status
Bartos et al., 2012	NfL	ELISA (Progen, Germany)	AD (25) PSP, FTD, CJD, CBS, WE (13)	73 (8) 64 (8)	21 (84%) 4 (23%)	N.R			MMSE (derived from ACE‐CZ), ACE‐CZ	None
Begcevic et al., 2020	NPTX1 NPTXR	Mass spectrometry	Cohort 1 (58): MCI (8) Mild AD (11) Moderate AD (24) Severe AD (15) Cohort 2 (43): MCI (6) Mild AD (8) Moderate AD (16) Severe AD (15)	74.5 (7.8) 71.4 (8.4) 75.7 (6.4) 74.4 (9.3) 67.6 (9.2) 76.2 (8.8) 78.1 (6.9) 71.1 (9.0)	3 (38%) 3 (27%) 13 (54%) 6 (40%) 5 (83%) 3 (38%) 6 (38%) 2 (15%)	N.R			MMSE	None
Bendlin et al., 2012	NfL	ELISA (in‐house)	CU with family history of AD (43)	53.67 (7.77)	31 (72.1%)	N.R			BVMT, COWAT, TMT‐A, TMT‐B, WAIS‐working memory index, AVLT	Age, education
Bjerke et al., 2009	NfL	ELISA (in‐house)	MCI‐SVD (9) MCI‐MD (15) MCI‐MCI (118) MCI‐AD (20) CU (52)	Median {25^th^, 75^th^ percentile} 68 {66, 74} 69 {65, 74} 62 {57, 68} 68 {58, 72} 66 {63, 70}	4 (44.4%) 13 (86.7%) 65 (55.1%) 12 (60%) 30 (57.7%)	Median {25^th^, 75^th^ percentile} 424 {255, 1414} 250 {250, 406} 250 {250, 250} 250 {250, 341} 250 {250, 250}			MMSE	None
Boiten et al., 2021	NPTX2	ELISA (in‐house)	AD (20) DLB (48)	65.3 (6.0) 67.7 (6.4)	2 (10%) 6 (13%)	Median [95% interval] 453 [317‐696] 474 [279 – 659]			Global, memory, attention, executive function, language, visual composites, MMSE	Age, education
Bos et al., 2019	NfL Ng	ELISA (UmanDiagnostics) Electrochemiluminescence (in‐house)	AD (180) Aβ + (157) Aβ ‐ (23) MCI (450) Aβ + (263) Aβ ‐ (187) CU (140) Aβ + (45) Aβ ‐ (95)	69.8 (8.8) 74.2 (7.9) 71.4 (7.1) 68.6 (8.2) 69.5 (8.1) 62.7 (7.3)	85 (54%) 8 (34%) 145 (55%) 89 (48%) 23 (51%) 49 (52%)	NfL 1742.2 (2893.2) 1931.9 (1934.8) 1242.3 (2556.1) 1031.2 (919.1) 983.13 (678.4) 627.4 (293.3	Ng 155.2 (121.4) 118.3 (136) 175.5 (217.8) 99.2 (102.9) 152.6 (149.6) 110.8 (224)		MMSE	Age, sex, years of education, baseline diagnosis
Brinkmalm et al., 2014	SNAP‐25	Mass spectrometry	AD (36) CU (33)	Median [IQR] Cohort 1: 68 [68‐79] Cohort 2: 77 [73‐82] Cohort 3: 68 [66‐70] Cohort 1: 70 [68‐74] Cohort 2: 54 [48‐63] Cohort 3: 66 [64‐68]	Cohort 1: 6 (66.7%) Cohort 2: 7 (70%) Cohort 3: 12 (70.6%) Cohort 1: 7 (77.8%) Cohort 2: 5 (83.3%) Cohort 3: 8 (47.1%)	N.R.			MMSE	None
Bruno et al., 2020	Ng Alpha‐Synuclein	ELISA (in‐house) ELISA (Tecan Sunrise, Austria)	CU (19)	68.1 (7.3)	12 (63%)	Ng: 100.8 (91.4)	α‐syn: 14.1 (16.1)		BSRT	None
Casaletto et al., 2017	Ng	ELISA (in‐house)	CU with family history of dementia (132)	64.5 (7.4)	86 (65.2%)	Median [IQR] 335.9 [250.6‐482.8]			AVLT, WAIS‐III symbol digit coding, BNT, WAIS‐III digit span forwards, WAIS‐III digit span backwards.	Sex CSF Aβ^42^ CSF t‐tau CSF p‐tau Hippocampal volume ApoE status Family history of AD
Chatterjee et al., 2018	Contactin‐2	ELISA (R&D, USA)	AD (106) CU (48)	Cohort 1: 62 (6) Cohort 2: 62 (5) Cohort 1: 60 (7) Cohort 2: 62 (3)	21 (58.3%) 41 (58 %) 15 (53.6%) 6 (30.6%)	Median [IQR] 59 [42‐74] 61 [39‐78] 78 [69‐110] 65 [54‐99]			MMSE	None
De Vos et al., 2016	Ng	ELISA (in‐house)	AD (50) MCI (38)	Median {25^th^, 75^th^ percentile} 75 {68, 78} 73 {69, 79}	27 (54%) 23 (60.5%)	Median [IQR] 172 [141‐230] 214 [161‐256]			MMSE	Age Sex
De Jong et al., 2007	NfL	ELISA (in‐house)	EAD (37) LAD (33) DLB (18) FTD (28)	Median [IQR] 61 [52‐69] 76 [69‐90] 72 {58‐90] 63 [43‐79]	22 (59.4%) 20 (60.6%) 5 (27.8%) 8 (28.6%)	Median [range] 6.1 [0.0‐40.3] 15.2 [0.0‐70.1] 10.4 [0.0‐60.4] 16.9 [0.0‐76.4]			MMSE	None
Delaby et al., 2020	NfL	ELISA (UmanDiagnostics, Sweden)	CU (118) AD (116) FTD (56) DLB (37) Prodromal DLB (26) PSP (12) CBS (26)	59.4 (9.7) 70.4 (8.0) 65.8 (5.2) 76.7 (4.9) 82.2 (6.1) 70.5 (7.8) 72 (7.3)	68 (57.6%) 71 (61.2%) 15 (26.8%) 19 (51.4%) 13 (50%) 7 (58.3%) 13 (50%)	Median [IQR] 411 [343‐567] 940 [765‐1229] 1240 [859‐2378] 1135 [803‐1321] 934 [643‐1094] 1422 [1034‐1727] 1637 [923‐2797]			MMSE	None
Dhiman et al., 2020	NfL	ELISA (UmanDiagnostics, Sweden)	AD (28) MCI (34) CU (159)	74.6 (7.5) 74.1 (7.6) 72.8 (5.5)	12 (43%) 13 (38%) 84 (53%)	2201 (626.96) 1977 (908.44) 1506 (510.59)			MMSE	Age Sex ApoE status
Galasko et al., 2019	Ng (Cohort 1) SNAP‐25 (Cohort 1) NPTX2 (Cohort 1, Cohort 2)	ELISA (EUROIMMUN, Germany) SIMOA (home‐brew) ELISA (in‐house)	Cohort 1 (193): AD MCI CU Cohort 2 (292): AD MCI CU	70.7 (9.4) 74.3 (6.5) 73 (5.2) 75.1 (7.6 74.7 (7.2) 75.7 (5.5)	19 (41%) 20 (35%) 52 (35%) 28 (42%) 44 (31%) 43 (50%)	Ng 347.6 (235.6) 332.2 (199.9) 324.5(163.4)	SNAP‐25 36 (15.6) 34.9(15.5) 32.1( 9.8)	NPTX2 715.1 (426.6) 826.5 (474.4) 1075 (504.8) 10.3 (0.9) 10.6 (0.7) 10.7 (0.5).	CVLT	Age Sex Education ApoE status
Gifford et al., 2018	NfL	ELISA (UmanDiagnostics, Sweden)	Early MCI (9) MCI (37) CU (65)	72 (7) 74(7) 73 (7)	2 (22%) 13 (35%) 20 (31%)	1145 (477) 1395 (795) 959 (466)			PVLT	Age, sex, ethnicity, ApoE status, cognitive diagnosis
Headley et al., 2018	Ng	Electrochemiluminescence (Meso Scale Discovery, USA)	MCI (193) CU (111)	75 (7) 75 (6)	64 (33%) 55 (50%)	494 (353) 352 (294)			MMSE, ADAS‐Cog, ADAS‐Cog13, memory composite, executive function composite	Age, sex, years of education, ApoE status, CSF t‐tau, CSF Aβ^42^
Hellwig et al., 2015	Ng	Electrochemiluminescenc (Meso Scale Discovery, USA)	AD (39) MCI‐AD (13) Non‐AD dementia (14) MCI‐O (29)	Median (range) 72.5 (68‐76) 73.3 (69‐77) 65.1 (59‐71) 69.4 (61‐75)	21 (53.9%) 8 (61.5%) 8 (57.1%) 14 (48.3%)	N.R			MMSE	None
Hoglund et al., 2015	NfL Ng VILIP‐1	ELISA (UmanDiagnostics, Sweden) Electrochemiluminescence (Meso Scale Discovery, USA) ELISA (BioVendor R&D, Germany)	CU Aβ‐ (43) CU Aβ+ (86)	*Total:* 81.9 (3.4)	*Total:* 73 (56.6%)	NfL 1847 (987.2) 1940 (1353)	Ng 889.3 (414.5) 686.1 (322.8)	VILIP‐1 0.13 (0.06) 0.12 (0.05)	MMSE	N.R
Jia et al., 2020	Ng GAP‐43 SNAP‐25 Synaptotagmin‐1	ELISA (American Research Products, USA) ELISA (MyBiosource, USA) ELISA (Proteintech, USA) ELISA (Abbkine, China)	Cohort 1: AD (28) Cohort 2: AD (73)	66 (6) 65 (6)	16 (57.1%) 42 (57.5%)	N.R			MMSE	Age, sex, ApoE status
Kirsebom et al., 2018	Ng	ELISA (EUROIMMUN, Germany)	Aβ+ MCI (20) Aβ+ SCI (18) CU (36)	66.8 (7.4) 66.7 (6.8) 61.2 (9.2)	12 (57%) 8 (44%) 19 (52.8%)	428 (179) 468 (217) 374 (128)			MMSE CERAD word list test, TMT‐A, TMT‐B	Age
Kvartsberg et al., 2015	Ng	ELISA (in‐house)	MCI (40)	Median [IQR] 64 [58‐71]	19 (48%)	Median [IQR] 210 [83‐433]			MMSE	Age, sex
Lee et al., 2008	VILIP‐1	ELISA (in‐house)	AD (33)	Mean ± SE 67.0 ± 1.8	18 (55%)	N.R			MMSE	None
Lim et al., 2019	NPTXR	ELISA (RayBiotech, USA)	MCI (14) Mild AD (21) Moderate AD (43) Severe AD (30)	72.1 (9.3) 73.7 (8.5) 77.0 (9.0) 72.8 (9.6)	8 (57%) 6 (29%) 19 (44%) 9 (30%)	N.R			MMSE	None
Mattsson et al., 2016	NfL Ng	ELISA (UmanDiagnostics, Sweden) ELISA (in‐house)	AD MCI CU	74.7 (8) 74.5 (7.5) 75.7 (5.2)	41 (44%) 62 (33%) 54 (50%)	N.R			MMSE, ADAS‐Cog11	Age, sex, years of education
McGuire et al., 2015	NfL pNfH	ELISA (UmanDiagnostics, Sweden ELISA (BioVendor,Czech Republic)	HAD (3) ANI (15) MNCD (15) CU (15)	Median [IQR] 47 [38‐50] 38 [31‐40] 40 [35‐48] 44 [36‐49]	0 (0%) 6 (40%) 3 (20%) 3 (20%)	N.R			WAIS‐III Digit symbol WAIS‐III Symbol search TMT‐A Story memory test Figure memory test WCST TMT‐B COWAT ANT WAIS‐III letter‐number sequencing PASAT	None
Meeter et al., 2016	NfL	ELISA (UmanDiagnostics, Sweden)	FTD with *GRN, MAPT, C9orf72* mutation (101)	Median [IQR] 59 [56‐65]	52 (51%)	6762 (N.R)			MMSE	None
Meeter et al., 2018	NfL	ELISA (UmanDiagnostics)	FTD with *C9orf72* mutation (64) Presymptomatic carriers of *C9orf72* mutation (25)	Median [IQR] 60 [55‐66] 47 [41‐57]	29 (45.3%) 17 (68%)	Median [IQR] 1885 [848‐2841] 429 [336‐830]			MMSE	None
Meeter et al., 2019	NfL	ELISA (UmanDiagnostics, Sweden)	svPPA (147)	Median [IQR] 64 [58‐68]	87 (54%)	Median [IQR] 2326 [1628‐3593]			BNT, ANT, letter fluency, WAIS‐III digit span forward and backwards, TMT‐A, TMT‐B, SCWT, CDT, AVLT, CVLT, CERAD word list test, Rey complex figure test	Age, sex, laboratory
Meeter et al., 2017	NfL	ELISA (UmanDiagnostics)	bvFTD (164) svPPA (36) nfvPPA (19) lvPPA (4) CBS (40) PSP (58)	Median [IQR] 61 [55‐67] 62 [58‐65] 62 [52‐66] 64 [51‐69] 65 [60‐73] 66 [62‐70]	78 (44%) 10 (53%) 10 (53%) 3 (75%) 14 (33%) 36 (56%)	Median [IQR] 3168 [1752‐4818] 3151[1906=4802] 2345 [1956‐2957] 1731 [1181‐2472] 2664 [1715‐4158] 1907 [1474‐2755]			MMSE, FAB	None
Mielke et al., 2019a	NfL Ng	ELISA (in‐house) ELISA (in‐house)	Dementia MCI CU *Total (777)*	Median [IQR] *Total* = 72.9 [64‐79.3]	*Total =* 334 (43%)	NfL (total) 520.2 [374.3‐745.4]	Ng (total) 166.6 [132.9‐220.8]		Global, Memory, language, attention, visuospatial composites	Age, sex
Mielke et al., 2019b	NfL	ELISA (in‐house)	MCI CU *Total (79)*	Median [IQR] *Total =* 76.4 [71.7‐80.7]	*Total* = 27 (34%)	Median [IQR] *Total* = 608.3 [429.1‐817.7]*			Memory, language, executive function, visuospatial composites	Age, sex, years of education
Mouton‐Liger et al., 2020	Nrg1	ELISA (R&D Systems, USA)	AD (54) MCI‐AD (20) Non‐AD dementia (30) Non‐AD MCI (31) CU (27)	69.4 (7.9) 70.2 (8.0) 68.7 (7.6) 61.5 (9.6) 62 (11.3)	33 (61.1%) 12 (60%) 11 (36.7%) 11 (35.5%) 23 (85.2%)	364.7 (149.2) 342.6 (161.5) 287.5 (106.5) 304.9 (113.0) 267.7 (104.2)			MMSE	None
Oeckl et al., 2020	Beta‐synuclein	Mass spectrometry	Cohort 1: AD (64) Cohort 2: AD (40) Cohort 3: AD (49)	Median [IQR] 73 [68‐78] 70 [63‐74] 72 [64‐77]	42 (65.6%) 20 (50%) 25 (51.0%)	Median [IQR] 979 [738‐1223] 694 [532‐990] 917 [746‐1185]			MMSE	None
Öhrfelt et al., 2016	Synaptotagmin	Mass spectrometry	Cohort 1: AD (17) Cohort 2: AD (24) Cohort 1: MCI‐AD (5) Cohort 2: MCI‐AD (18) Cohort 1: CU (17) Cohort 2: CU (36)	Median [IQR] 65 [58‐81] 68 [64‐72] 78 [73‐81] 70 [69‐78] 60 [53‐67] 62 [55‐69]	12 (70.6%) 17 (70.8%) 4 (80%) 13 (72.2%) 10 (58.8%) 23 (63.9%)	N.R			MMSE	None
Öhrfelt et al., 2019	SNAP‐25	ELISA (in‐house)	Cohort 1: AD (17) Cohort 2: AD (24) Cohort 1: MCI‐AD (5) Cohort 2: MCI‐AD (18) Cohort 1: CU (17) Cohort 2: CU (36)	Median [IQR] 65 [58‐81] 68 [64‐72] 78 [73‐81] 70 [69‐78] 60 [53‐67] 62 [55‐69]	12 (70.6%) 17 (70.8%) 4 (80%) 13 (72.2%) 10 (58.8%) 23 (63.9%)	N.R			MMSE	None
Osborn et al., 2019	NfL	ELISA (UmanDiagnostics, Sweden)	Early MCI (27) MCI (132) CU (174)	73 (6) 73 (8) 72 (7)	7 (26%) 58 (44%) 71 (41%)	1088 (465) 1250 (712) 930 (448)			Episodic memory composite, executive function composite, BNT, ANT, WAIS‐IV coding, DKEFS number sequencing, Hooper visual organisation test	Age, sex, ethnicity, ApoE status
Portelius et al., 2015	Ng	Electrochemiluminescence (in‐house)	AD (95) pMCI (105) sMCI (68) CU (110)	Median [IQR] 76 [70‐80] 75 [70‐80] 74 [70‐80] 76 [72‐78]	42 (44%) 37 (35%) 22 (32%) 55 (50%)	Median [IQR] 485 [349‐744]* 492 [330‐672]* 386 [190‐582]* 304 [161‐453]*			MMSE, ADAS‐Cog	Age, sex, education
Racine et al., 2016	NfL	ELISA (UmanDiagnostics, Sweden)	MCI + CU (70)	66.26 (6.1)	40 (57.1%)	N.R			CAB CPAL errors GMCT moves/sec GML errors GMR errors OCL accuracy ONB accuracy TWOB accuracy RAVLT delayed Logical memory delayed BVMT‐R delayed	None
Rojas et al., 2018	NfL	ELISA (UmanDiagnostics, Sweden)	PSP (50)	67.7 (5.7)	30 (60%)	5929 (6196)			RBANS Color trails 1 & 2 Letter‐number sequencing, Phonemic fluency	Age, sex
Rolstad et al., 2015a	NfL	ELISA (in‐house)	Dementia‐ vascular (65) Dementia‐ non‐vascular (128) MCI‐ vascular (86) MCI‐ non‐vascular (175) SCI‐ vascular (48) SCI‐ non‐vascular (120)	68.9 (6.5) 66.4 (7.8) 67.4 (7.2) 63.9 (7.7) 65.6 (7.4) 60.6 (7.1)	32 (49.2%) 78 (60.9%) 50 (58.1%) 60 (34.3%) 28 (58.3%) 72 (60%)	567.5 (635.0) 569.4 (720.3) 611.2 (1110.9) 360.7 (299.6) 308.5 (158.2) 328.3 (295.8)			Attention, learning/memory, visuospatial, language, executive function composites,	Age, sex
Rolstad et al., 2015b	NfL	ELISA (UmanDiagnostics)	CU (71)	37.8 (14.6)	44 (61.9%)	254.38 (55.42)			Memory , executive function, visuospatial, speed/attention, verbal composites	Age, sex
Sancesario et al., 2020	Ng	ELISA (EUROIMMUN, Germany)	CU (30)	64.04 (11.83)	18 (61%)	336.53 (193.40)			MMSE	None
Sandelius et al., 2019	GAP‐43	ELISA (in‐house)	AD (275) MCI (84) CU (43) FTD (39) DLB (27) lvPPA (10) svPPA (15) PSP (18) CBS (19)	71.2 (9.2) 72 (8.9) 69 (9.1)	58.2% 46.4% 69.8%	N.R			MMSE	None
Sanfilippo et al., 2016	Ng	ELISA (in‐house)	AD (25) MCI (50) MCI‐AD (36) CU (44)	Median [IQR] 76 [67‐85] 71 [68‐76] 73 [71‐76] 71 [67.5‐75]	19 (76%) 30 (60%) 22 (61%) 31 (70.5%)	Median [IQR] 687 [474‐956]* 182 [83‐310]* 481 [326‐841]* 235.5 [171‐358]*			MMSE, CAMCOG	None
Santillo et al., 2019	Ng	Electrochemiluminescence (Meso Scale Discovery, USA)	CU (20)	25 (4)	9 (45%)	427 (189)			MCCB	None
Scherling et al., 2014	NfL	ELISA (UmanDiagnostics, Sweden)	Asymptomatic FTD mutation carriers (8) bvFTD (45) nfvPPA (18) svPPA (16) CBS (17) AD (50) PSP (22) CU (47)	54 (10) 61 (8) 70 (7) 63 (7) 68 (8) 66 (9) 68 (7) 66 (11)	4 (100%) 13 (28.9%) 7 (38.9%) 10 (62.5%) 11 (64.7%) 22 (44%) 11 (50%) 21 (44.7%)				MMSE, Rey‐Osterrieth figure, FDS, BDS, TMT, Stroop task, BNT, ANT, CVLT, phonemic fluency	None
Schindler et al., 2019	Ng SNAP‐25 VILIP‐1	SIMOA (Millipore, USA) SIMOA (Millipore, USA) SIMOA (Millipore, USA)	Carriers of mutations in *PSEN1, PSEN2,* or *APP* (235) Mutation non‐carriers (145)	38.4 (10.4) 38.8 (12.1)	127 (54%) 89 (61%)	Ng 2269 (1189) 1572 (741)	SNAP‐25 4.6 (1.9) 3.7 (1.3)	VILIP‐1 173.4 (77.9) 132.9 (50.2)	DIAN cognitive composite	Age, sex, education, ApoE status
Sjögren et al., 2001	NfL	ELISA (in‐house)	AD (22) SVD (9) CU (20)	64.4 (7.7) 70.1 (6.3) 66.4 (9.9)	7 (31.8%) 9 (100%) 15 (75%)	569 (308) 1977 (1436) 156 (66)			MMSE	None
Sjögren et al., 2000	NfL	ELISA (in‐house)	FTD (18) AD (21)	62.4 (10) 73.4 (3.2)	7 (38.9%) 14 (66.7%)	1442 (1183) 1006 (727)			MMSE	None
Skillback et al., 2014	NfL	ELISA (UmanDiagnostics, Sweden)	EAD (223) AD (1194) FTD (146) DLB (114) VaD (465) MIX (517) PDD (45) Dementia NOS (437)	59 (4) 76 (6) 68 (9) 73 (7) 76 (8) 78 (7) 70 (8) 74 (9)	*Total* = 54.4%	448 (415) 667 (664) 1220 (1026) 622 (1217) 1059 (1207) 928 (1056) 503 (374) 807 (1237)			MMSE	Age, sex
Sun et al., 2016	Ng	Electrochemiluminescence (Meso Scale Discovery, USA)	ApoE ε4 carriers: AD (67) MCI (102) CU (27)	75 (8) 74 (8) 76 (5)	42 (44%) 64 (33%) 55 (50%)	N.R			MMSE	None
Swanson et al., 2016	NPTX2	Mass spectrometry	AD (64) MCI (135) CU (86)	74.98 (7.57) 74.69 (7.35) 75.70 (5.54)	29 (45.3%) 44 (32.6%) 42 (48.8%)	Mean ± SE 10.31 ± 0.09 10.62 ± 0.06 10.70 ± 0.08			MMSE, ADAS‐Cog, memory composite	Age, sex, education, ApoE status
Teitsdottir et al., 2020	NfL	ELISA (UmanDiagnostics, Sweden)	AD CSF profile (28) SCI (2) MCI (9) AD (16) DLB (1) Non‐AD CSF profile SCI (10) MCI (13) DLB (1)	Median (range) 70 (51‐84) 67 (46‐80)	11 (39.3%) 8 (80%)	Median (range) 2500 (1200 – 4500) 1900 (900 – 6500)			Verbal episodic memory composite	Age, education
Van Der Ende et al., 2020	NPTX2	ELISA (in‐house)	Symptomatic genetic FTD (54) Presymptomatic genetic FTD (106)	Median [IQR] 63 [56‐69] 45 [34‐56]	22 (40.7%) 59 (55.7%)	Median [IQR] 643 [301‐872] 1003 [624‐1358]			MMSE, TMT‐B, phonemic verbal fluency	Age, sex, years of education, study site
Van Steenoven et al., 2020	NPTX2 NPTXR	Mass spectrometry Mass spectrometry	Cohort 1: DLB (20) Cohort 2: DLB (17) Cohort 3: DLB (48)	65.3 (5.8) 66.9 (7.5) 67.8 (6.3)	3 (15%) 4 (24%) 6 (12%)	N.R			MMSE	Cohort
Wang et al., 2019	Ng	Electrochemiluminescence (Meso Scale Discovery, USA)	AD (81) MCI (171) CU (99)	74.6 (7.8) 74.2 (7.6) 75.5 (5.3)	37 (45.7%) 58 (33.9%) 49 (49.5%)	Median [IQR] 471 [347‐675] 455 [267‐657] 324 [191‐468]			MMSE	None
Wang et al., 2018	SNAP‐25	ELISA (Erenna, USA)	AD (16) MCI (75) CU (55)	73.4 (6.8) 74.3 (6.5) 76 (5)	10 (62.5%) 21 (28%) 24 (43.6%)	N.R			MMSE	None
Wellington et al., 2016	Ng	Electrochemiluminescence (in‐house)	AD (100) Genetic AD (2) bvFTD (20) svFTD (21) LBD (13) PSP (46) CU (19)	Median [IQR] 63 [57‐68] 43, 47 61 [57‐69] 69 [61‐73] 68 [66‐76] 70 [66‐72] 61 [50‐64]	59 (59%) 2 (100%) 8 (40%) 10 (48%) 2 (15%) 19 (41%) 11 (58%)	Median [IQR] 463 [275‐669] 252, 1162 150 [120‐317] 244 [138‐426] 120 [120‐304] 188 [120‐302] 196 [120‐297]			MMSE	None
Xiao et al., 2017	NPTX2	ELISA (in‐house)	AD (30)	Mean ± SE 72.24 ± 10.15	16 (53.3%)	Mean ± SE 716.12 ± 388.22			MMSE, DSS, BNT, phonemic verbal fluency, semantic verbal fluency, Wisconsin card sorting task, visual reproduction test, block design, CDT, CVLT	None
Zetterberg et al., 2016	NfL	ELISA (UmanDiagnostics, Sweden)	AD (95) pMCI (101) sMCI (91) CU (110)	Median [IQR] 76 [69‐80] 74 [69‐80] 74 [71‐80]	42 (44.2%) 37 (36.6%) 26 (28.6%)	Median [IQR] 1479 [1134‐1842] 1336 [1061‐1693] 1182 [923‐1687]			MMSE, ADAS‐Cog	Age, sex, education
Zhang et al., 2018a	VILIP‐1	ELISA (Erenna, USA)	AD (18) sMCI (24 pMCI (47) CU (32)	74.3 (6.79) 76.7 (5.34) 73.1 (6.86) 76 (5.66)	11 (61.1%) 7 (29.2%) 14 (29.8%) 13 (40.6%)	189.7 (70.43) 146 (51.93) 184.3 (64.44) 133.0 (37.9)			MMSE	Age, sex, education
Zhang et al., 2018b	SNAP‐25	ELISA (Erenna USA)	AD (18) sMCI (22) pMCI (47) CU (52)	74.3 (7) 76 (5.1) 73.1 (6.6) 76.2 (5.1)	11 (61.1%) 7 (31.8%) 14 (29.8%) 22 (42.3%)	N.R			MMSE1	Age, sex, education

* Age and CSF levels presented as mean (SD) unless otherwise specified

ACE‐CZ, Addenbrooke’s Cognitive Examination‐ Czech Version; AD, Alzheimer’s Disease; ADAS‐Cog, Alzheimer Disease Assessment Scale cognitive subscale; ALS, Amyotrophic Lateral Sclerosis; ANI, Asymptomatic Neurocognitive Impairment; ANT, Animal Naming Test; ApoE, Apolipoprotein E; AVLT, Rey Auditory Verbal Learning Test; Aβ‐, Amyloid beta negative; Aβ+, Amyloid beta positive; BMDB, Brief Mental Deterioration Battery; BNT, Boston Naming Test; BSRT, Buschke Selective Reminding Test; bvFTD, Behaviour Variant FTD; BVMT‐R, Brief Visuospatial Memory Test‐ Revised; BVMT, Brief Visuospatial Memory Test; CAMCOG, Cambridge Cognitive Examination; CBS, Corticobasal Syndrome; CDT, Clock Drawing Test; CERAD, Consortium to Establish a Registry for Alzheimer’s Disease; CJD, Creutzfeldt‐Jacob Disease; COWAT, Controlled Oral Word Association Test; CPAL, Continuous Paired Associate Learning; CU, Cognitive unimpaired; CVLT, California Verbal Learning Test; DIAN, Dominantly Inherited Alzheimer Network; DKEFS, Delis‐Kaplan Executive Function System; DLB, Dementia with Lewy Bodies; DSB, Digit Span Backwards; DSF, Digit Span Forwards; DSS, Digit Symbol Substitution; EAD, Early onset Alzheimer’s Disease; FAB, Frontal Assessment Battery; FTD, Frontotemporal Dementia; GMCT, Groton Maze Times Chase Test; GML, Groton Maze Learning Test; GMR, Groton Maze Learning Test delayed recall; HAD, HIV‐Associated Dementia; lvPPA, logopenic variant Primary Progressive Aphasia; MCCB, MATRICS Consensus Cognitive Battery; MCI‐AD, Mild Cognitive Impairment due to Alzheimer’s Disease; MCI‐o, Mild Cognitive Impairment not due to Alzheimer’s Disease; MCI, Mild Cognitive Impairment; MIX, Mixed Dementia; MMSE, Mini Mental State Examination; MNCD, Mild Neurocognitive Disorder; MND, Motor Neuron Disease; MSA, Multiple System Atrophy; NfL, Neurofilament‐Light; nfvPPA, non‐fluent variant Primary Progressive Aphasia; Ng, Neurogranin; NOS, Not Otherwise Specified; OCL, One‐Card Learning; ONB, One‐Back Memory; PASAT, Paced Auditory Serial Addition Test; PCA, Posterior Cortical Atrophy; PD, Parkinson’s Disease; PDD, Parkinson’s Disease Dementia; pDLB, Prodromal Dementia with Lewy Bodies; pMCI, progressive MCI; pNfH, Phosphorylated Neurofilament Heavy; PSP, Progressive Supranuclear Palsy; PVLT, Philadelphia Verbal Learning Test; RBANS, Repeatable Battery for the Assessment of Neuropsychological Status; SCWT, Stroop Color Word Test; sMCI, stable MCI; SVD, Small Vessel Disease; svPPA, Semantic Variant Primary Progressive Aphasia; TMT‐ B, Trail Making Test B; TMT‐A, Trail Making Test A; TWOB, Two‐Back Memory; VaD, Vascular Dementia; WAIS, Wechsler Adult Intelligence Scale; WCST, Wisconsin Card Sorting Test; WE, Wernicke’s Encephalopathy

Sample sizes of included studies ranged from 19 to 770. Only one of the included studies conducted a power analysis (Xiao et al., 2017), although others acknowledged a possible lack of power.

#### 3.2.2 Sociodemographic factors

Participants with AD were aged between 62 and 77 years, those with FTD were aged between 59 and 72 years and MCI participants' age ranged from 62 to 76 years. The age ranges of participants are within the typical range for the detection of dementia/MCI‐related cognitive decline. Those with an ‘other’ form of dementia were aged between 39.5 and 76.7 years. CU participants' age varied widely (between 37.8 and 81.9) due to the nature of the healthy ageing groups; some findings were taken from studies investigating neurodegenerative diseases with age‐matched controls, while few focused solely on CU younger participants. Most studies included a mix of both males and females.

#### 3.2.3 Group status and dementia definitions

As reported in Table 1, 46 studies included participants with AD or MCI, 15 examined those with an FTD‐related syndrome, 39 examined controls or CU samples and 9 studies included those with an ‘other’ dementia. All studies used validated criteria for diagnosing dementia, MCI or identifying the absence of dementia. In AD, most studies used the National Institute of Neurological and Communicative Disorders and the Alzheimer's Disease and Related Disorders Association (NINCDS‐ADRDA) (McKhann et al., 1984) criteria to diagnose probable AD and others used the updated National Institute on Ageing/Alzheimer's Association (NIA‐AA) criteria (Jack et al., 2018). One study used The International Working Group 2 (IWG‐2) (Dubois et al., 2014) criteria, and in five, diagnoses were made by clinicians (which were supplemented with CSF marker information in two). To confirm familial AD, one study used autopsy and medical records matched with NINCDS‐ADRDA criteria, while another used autopsy records and the Kawas Dementia Questionnaire (Kawas et al., 1994). Studies with MCI patients used established criteria proposed by the IWG‐2 (Winblad et al., 2004), NIA‐AA criteria (Albert et al., 2011) or criteria proposed by Petersen and colleagues (Petersen, 2004). Two studies used criteria for early MCI proposed by Aisen and colleagues (Aisen et al., 2010) which defines early MCI as a milder episodic memory impairment relative to ‘late MCI’. All FTD studies used established criteria for the relevant subsyndrome, which were appropriate for the time of publication (Armstrong et al., 2013; Gorno‐Tempini et al., 2011; Litvan et al., 1996; Neary et al., 1998; Rascovsky et al., 2011). Most CU studies ruled out dementia or cognitive impairment if participants had a Clinical Dementia Rating (CDR) of 0 or did not meet DSM‐III‐R criteria.

#### 3.2.4 Adjustment factors

As seen in Table 1, adjustment factors varied between studies. Thirty‐four studies did not adjust for any covariates. One study conducted a partial correlation and adjusted for multiple cohorts (van Steenoven et al., 2020). Studies using regression techniques most often controlled for age, sex and years of education. Nine studies controlled for ApoE e4 status, and two controlled for ethnicity.

#### 3.2.5 Cognitive assessments

A number of tools were used to assess neuropsychological performance (Table 1). Including composite measures as single tests, there were 37 different cognitive tests analysed across all 67 studies. The most commonly used test was the Mini Mental State Examination (MMSE) (Folstein et al., 1975), which was employed in 48 studies. The main domains assessed were global cognition, visuospatial abilities, language, attention, general executive functions and memory (working, episodic and semantic).

#### 3.2.6 Risk of bias

Risk of bias ratings is provided in the supporting information Table S2. Twenty studies were rated as ‘Good’, 45 rated as ‘Fair’ and 3 as ‘Poor’.

#### 3.2.7 CSF markers

As reported in Table 1, most studies assayed multiple markers. Thirty‐one studies examined NfL, 22 examined Ng and 24 studies examined a different marker of interest. A description of each marker can be found in Table 2.

**TABLE 2:** Summary of CSF markers from included studies

CSF marker	Function	Localization
Alpha‐Synuclein	Regulation of synaptic vesicle trafficking	Pre‐synaptic
Beta‐Synuclein	Unknown	Pre‐synaptic
Contactin‐2	Axonal guidance Axonal fasciculation	Pre‐synaptic Axonal
GAP‐43	Axonal outgrowth Synaptic plasticity	Pre‐synaptic
NfH	Neuronal structure	Axonal
NfL	Neuronal structure	Axonal
Ng	Calmodulin‐binding LTP signalling	Post‐synaptic
NPTX1	Synaptic plasticity Facilitates excitatory synapse formation	Pre‐synaptic
NPTX2	Synaptic plasticity Facilitates excitatory synapse formation	Pre‐synaptic
NPTXR	Synaptic plasticity Facilitates excitatory synapse formation	Trans‐synaptic
Nrg1	Synaptic plasticity	Pre‐synaptic
SNAP‐25	SNARE	Pre‐synaptic
Synaptotagmin	Calcium sensor	Pre‐synaptic
VILIP‐1	Calcium sensor	Neuronal

Abbreviations: CSF, cerebrospinal fluid; GAP‐43, growth‐associated protein 43; NfH, neurofilament‐heavy; NfL, neurofilament‐light; Ng, neurogranin; NPTX1, neuronal pentraxin I; NPTX2, neuronal pentraxin 2; NPTXR, neuronal pentraxin receptor; Nrg1, neuregulin‐1; SNAP‐25, synaptosomal‐associated protein 25; VILIP‐1, visinin‐like protein‐1.

A number of immunoassay methods were used to measure CSF analytes. Enzyme‐linked immunosorbent assays (ELISAs) were the most common immunoassay method, followed by electrochemiluminescence and mass‐spectrometry based methods. Two studies used SIMOA assays. Of the included 67 studies, only 29 reported the intra‐assay coefficient of variability (CV) (Abu‐Rumeileh et al., 2018; Bartos et al., 2011; Bendlin et al., 2012; Bjerke et al., 2009; Brinkmalm et al., 2014; Casaletto et al., 2017; Chatterjee, Del Campo, et al., 2018; Dhiman et al., 2020; Gifford et al., 2018; Hellwig et al., 2015; Hoglund et al., 2017; Kirsebom et al., 2018; Kvartsberg, Duits, et al., 2015; Lim et al., 2019; Meeter et al., 2016; Meeter, Gendron, et al., 2018; Meeter et al., 2019; Meeter, Vijverberg, et al., 2018; Mielke, Syrjanen, Blennow, Zetterberg, Vemuri, et al., 2019; Öhrfelt et al., 2019; Osborn et al., 2019; Rolstad, Jakobsson, et al., 2015; Sandelius et al., 2019; Skillback et al., 2014; Teitsdottir et al., 2020; van der Ende et al., 2020; Wellington et al., 2016; Zetterberg et al., 2016) and only 22 reported inter‐assay CVs (Abu‐Rumeileh et al., 2018; Bartos et al., 2011; Bjerke et al., 2009; Brinkmalm et al., 2014; Chatterjee, Del Campo, et al., 2018; Dhiman et al., 2020; Hellwig et al., 2015; Hoglund et al., 2017; Kvartsberg et al., 2015; Meeter et al., 2016; Meeter, Gendron, et al., 2018; Meeter et al., 2019; Meeter, Vijverberg, et al., 2018; Mielke, Syrjanen, Blennow, Zetterberg, Skoog, et al., 2019; Mielke, Syrjanen, Blennow, Zetterberg, Vemuri, et al., 2019; Mouton‐Liger et al., 2020; Rolstad, Jakobsson, et al., 2015; Sandelius et al., 2019; Singh et al., 2016; Teitsdottir et al., 2020; van der Ende et al., 2020); therefore, the repeatability and technical heterogeneity of results was not reported in the majority of studies.

### 3.3 Main outcome: Associations between CSF markers and neuropsychological performance

#### 3.3.1 Papers on CSF NfL

In total, 31 studies examined the relationship between CSF NfL levels and neuropsychological performance. All studies analysed CSF NfL using ELISAs.

As reported in Table 3, a significant association between CSF NfL and neuropsychological performance was consistently reported in AD samples. Most studies found significant moderate‐to‐weak relationships with MMSE scores (Abu‐Rumeileh et al., 2018; Bos et al., 2019; Delaby et al., 2020; Sjogren et al., 2000; Skillback et al., 2014; Zetterberg et al., 2016), while others showed no relationship (Bartos et al., 2011; de Jong et al., 2007; Rolstad, Berg, et al., 2015). However, sample sizes were relatively small in two of these studies. Only two studies included early‐onset Alzheimer's (EAD) samples, and both reported no significant associations with MMSE scores (de Jong et al., 2007; Skillback et al., 2014).

**TABLE 3:** Summary of results

Study	CSF marker	Population (*N*)	Cognitive assessment and direction of relationship ( 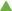 ‐positive relationship, 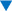 ‐ negative, * non‐significant; non‐adjusted results reported where available)	
Abu‐Rumeileh et al. (2018)	NfL	AD (60)	MMSE	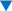
		FTD (141)	BMDB	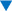
			FAB	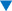
			MMSE	*
Agnello et al. (2020)	Ng	AD (29)	MMSE	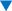
	Alpha‐synuclein	AD (29)	MMSE	*
Alcolea et al. (2017)	NfL	FTD (249)	MMSE	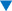
Aschenbrenner et al. (2020)	NfL	CU Aβ + (94) CU Aβ‐ (161)	Global cognition composite	*
			Episodic memory composite	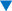
			Attention composite	*
Bartos et al. (2011)	NfL	AD (25)	MMSE (derived from ACE‐CZ)	*
			ACE‐CZ	*
		PSP, FTD, CJD, CBS, WE (13)	MMSE (derived from ACE‐CZ)	*
			ACE‐CZ	*
Begcevic et al. (2018)	NPTX1	Cohort 1 (58): MCI (8) Mild AD (11) Moderate AD (24) Severe AD (15)	MMSE	*
		Cohort 2 (43): MCI (6) Mild AD (8) Moderate AD (16) Severe AD (15)	MMSE	*
	NPTXR	Cohort 1 (58): MCI (8) Mild AD (11) Moderate AD (24) Severe AD (15)	MMSE	*
		Cohort 2 (43): MCI (6) Mild AD (8) Moderate AD (16) Severe AD (15)	MMSE	*
Bendlin et al. (2012)	NfL	CU with family history of AD (43)	BVMT	*
			COWAT	*
			TMT‐A	*
			TMT‐B	*
			WAIS‐working memory index	*
			AVLT	*
Bjerke et al. (2009)	NfL	MCI‐SVD (9) MCI‐MD (15) MCI‐MCI (118) MCI‐AD (20) CU (52)	MMSE	*
Boiten et al. (2021)	NPTX2	AD (20)	Global composite	*
			Memory composite	*
			Attention composite	*
			Executive function composite	*
			Language composite	*
			Visuospatial composite	*
			MMSE	*
		DLB (48)	Global composite	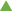
			Memory composite	*
			Attention composite	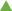
			Executive function composite	*
			Language composite	*
			Visuospatial composite	*
			MMSE	*
Bos et al. (2019)	NfL	Total Aβ + (465)	MMSE	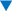
		Total Aβ‐ (305)		*
		AD (180)		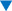
		MCI (450)		*
		CU (140)		*
	Ng	Total Aβ + (465) Total Aβ‐ (305)	MMSE	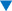 *
		AD (180)		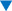
		MCI (450)		*
		CU (140)		*
Brinkmalm et al. (2014)	SNAP‐25	AD (36)	MMSE	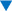
		CU (33)	MMSE	*
Bruno et al. (2020)	Alpha‐Synuclein	CU (19)	BSRT	*
	Ng	CU (19)	BSRT	*
Casaletto et al. (2017)	Ng	CU with family history of dementia (132)	AVLT	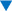
			WAIS‐III symbol digit coding	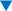
			BNT	*
			WAIS‐III DSF	*
			WAIS‐III DSB	*
Chatterjee, Del Campo, et al. (2018)	Contactin‐2	Total sample (154)	MMSE	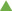
		AD (106)	MMSE	*
		CU (48)	MMSE	*
De Vos et al. (2016)	Ng	AD (50)	MMSE	*
		MCI (38)	MMSE	*
De de Jong et al. (2007)	NfL	EAD (37)	MMSE	*
		AD (33)	MMSE	*
		DLB (18)	MMSE	*
		FTD (28)	MMSE	*
Delaby et al. (2020)	NfL	CU (118)	MMSE	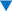
		AD (116)	MMSE	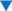
		FTD (56)	MMSE	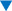
		DLB (37)	MMSE	*
		pDLB (26)	MMSE	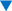
		PSP (12)	MMSE	*
		CBS (26)	MMSE	*
Dhiman et al. (2020)	NfL	Total sample (221) AD (28) MCI (34) CU (159)	MMSE	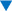
Galasko et al. (2019)	Ng	Total AD, MCI, CU (193)	CVLT immediate recall CVLT delayed recall	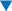 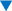
		Aβ/tau+	CVLT immediate recall CVLT delayed recall	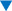 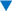
		Aβ/tau‐	CVLT immediate recall CVLT delayed recall	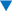 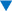
	NPTX2	Total AD, MCI, CU (193)	CVLT immediate recall CVLT delayed recall	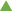 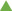
		Aβ/tau+	CVLT immediate recall CVLT delayed recall	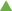 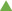
		Aβ/tau‐	CVLT immediate recall CVLT delayed recall	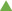 *
	SNAP‐25	Total AD, MCI, CU (193)	CVLT immediate recall CVLT delayed recall	* 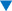
		Aβ/tau+	CVLT immediate recall CVLT delayed recall	* 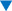
		Aβ/tau‐	CVLT immediate recall CVLT delayed recall	* *
Gifford et al. (2018)	NfL	Early MCI (9) MCI (37) CU (65)	PVLT List Total learning	*
			Short delay free recall	*
			Short delay cued recall	*
			Long delay free recall	*
			Long delay cued recall	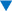
			Discrimination	*
		CU (65)	PVLT List Total learning	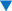
			Short delay free recall	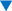
			Short delay cued recall	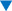
			Long delay free recall	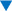
			Long delay cued recall	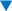
			Discrimination	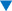
Headley et al. (2018)	Ng	MCI (193)	Memory composite	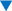
			Executive function composite	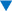
		CU (111)	Memory composite	*
			Executive function composite	*
		Total (304)	MMSE	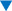
			ADAS‐cog	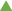
			ADAS‐Cog13	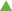
			Memory composite	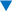
			Executive function composite	*
Hellwig et al. (2015)	Ng	AD + MCI‐AD (53)	MMSE	*
		Non‐AD dementia + MCI‐o (43)	MMSE	*
Hoglund et al. (2017)	NfL	CU Aβ‐ (43)	MMSE	*
		CU Aβ + (86)	MMSE	*
	Ng	CU Aβ‐ (43)	MMSE	*
		CU Aβ + (86)	MMSE	*
	VILIP‐1	CU Aβ‐ (43)	MMSE	*
		CU Aβ + (86)	MMSE	*
Jia et al. (2020)	Ng	Discovery cohort AD (28)	MMSE	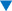
		Validation cohort (73)		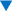
	GAP‐43	Discovery cohort AD (28)	MMSE	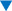
		Validation cohort (73)		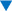
	SNAP‐25	Discovery cohort AD (28)	MMSE	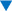
		Validation cohort (73)		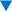
	Synaptotagmin‐1	Discovery cohort AD (28)	MMSE	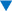
		Validation cohort (73)		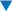
Kirsebom et al. (2018)	Ng	Aβ + MCI (20) Aβ + SCI (18) CU (36)	MMSE	*
			CERAD word list test	*
			TMT‐A	*
			TMT‐B	*
Kvartsberg, Duits, et al. (2015)	Ng	MCI (40)	MMSE	*
Lee et al. (2008)	VILIP‐1	AD (33)	MMSE	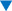
Lim et al. (2019)	NPTXR	MCI (14) Mild AD (21) Moderate AD (43) Severe AD (30)	MMSE	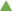
Mattsson et al. (2016)	NfL	Aβ + AD, MCI, CU (262)	MMSE	*
			ADAS‐Cog11	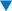
		Aβ‐ AD, MCI, CU (127)	MMSE	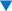
			ADAS‐Cog11	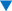
	Ng	Aβ + AD, MCI, CU (262)	MMSE	*
			ADAS‐Cog11	*
		Aβ‐ AD, MCI, CU (127)	MMSE	*
			ADAS‐Cog11	*
McGuire et al. (2015)	NfL	HAD (3) ANI (15) MNCD (15) CU (15)	WAIS‐III digit symbol	*
			WAIS‐III symbol search	*
			TMT‐A	*
			Story memory test	*
			Figure memory test	*
			WCST	*
			TMT‐B	*
			COWAT	*
			ANT	*
			WAIS‐III letter‐number sequencing	*
			PASAT	*
	pNfH	HAD (3) ANI (15) MNCD (15) CU (15)	WAIS‐III digit symbol	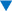
			WAIS‐III symbol search	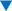
			TMT‐A	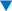
			Story memory test	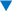
			Figure memory test	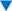
			WCST	*
			TMT‐B	*
			COWAT	*
			ANT	*
			WAIS‐III letter‐number sequencing	*
			PASAT	*
Meeter et al. (2016)	NfL	FTD with *GRN, MAPT, C9orf72* mutation (101)	MMSE	*
Meeter, Gendron, et al. (2018)	NfL	FTD with *C9orf72* mutation (64)	MMSE	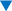
		Presymptomatic carriers of *C9orf72* mutation (25)	MMSE	*
		Total (89)	MMSE	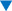
Meeter et al. (2019)	NfL	svPPA (147)	BNT	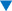
			ANT	*
			Letter fluency	*
			WAIS‐III DSF	*
			WAIS‐III DSB	*
			TMT‐A	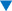
			TMT‐B	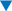
			SCWT	*
			CDT	*
			AVLT	*
			CVLT	*
			CERAD word list test	*
			Rey complex figure test	*
Meeter, Vijverberg, et al. (2018)	NfL	bvFTD (164)	MMSE	*
			FAB	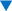
		svPPA (36)	MMSE	*
			FAB	*
		nfvPPA (19)	MMSE	*
			FAB	*
		lvPPA (4)	MMSE	*
			FAB	*
		CBS (40)	MMSE	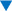
			FAB	*
		PSP (58)	MMSE	*
			FAB	*
		Total sample (including FTD‐MND;	MMSE	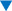
			FAB	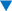
Mielke, Syrjanen, Blennow, Zetterberg, Skoog, et al. (2019)	NfL	Dementia (7) MCI (83)	Global composite	*
			Memory composite	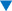
			Language composite	*
			Attention composite	*
			Visuospatial composite	*
		CU (687)	Global composite	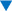
			Memory composite	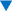
			Language composite	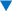
			Attention composite	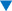
			Visuospatial composite	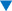
		*Total (777)*	Global composite	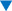
			Memory composite	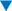
			Language composite	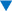
			Attention composite	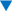
			Visuospatial composite	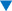
	Ng	Dementia (7) MCI (83)	Global composite	*
			Memory composite	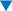
			Language composite	*
			Attention composite	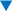
			Visuospatial composite	*
		CU (687)	Global composite	*
			Memory composite	*
			Language composite	*
			Attention function composite	*
			Visuospatial composite	*
		*Total (777)*	Global composite	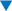
			Memory composite	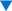
			Language composite	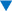
			Attention composite	*
			Visuospatial composite	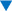
Mielke, Syrjanen, Blennow, Zetterberg, Vemuri, et al. (2019)	NfL	MCI (15) CU (64) *Total (79)*	Global composite	*
			Memory composite	*
			Language composite	*
			Attention composite	*
			Visuospatial composite	*
Mouton‐Liger et al. (2020)	Nrg1	AD (54)	MMSE	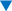
		MCI‐AD (20)	MMSE	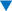
		Total: AD (54) MCI‐AD (20) Non‐AD dementia (30) Non‐AD MCI (31) CU (27)	MMSE	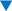
Oeckl et al. (2020)	Beta‐synuclein	Cohort 1: AD (64)	MMSE	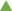
		Cohort 2: AD (40)	MMSE	*
		Cohort 3: AD (49)	MMSE	*
Öhrfelt et al. (2016)	Synaptotagmin	Cohort 1: AD (17)	MMSE	*
		Cohort 2: AD (24)	MMSE	*
		Cohort 1: MCI‐AD (5)	MMSE	*
		Cohort 2: MCI‐AD (18)	MMSE	*
		Cohort 1: CU (17)	MMSE	*
		Cohort 2: CU (36)	MMSE	*
Öhrfelt et al. (2019)	SNAP‐25	Cohort 1: AD (17)	MMSE	*
		Cohort 2: AD (24)	MMSE	*
		Cohort 1: MCI‐AD (5)	MMSE	*
		Cohort 2: MCI‐AD (18)	MMSE	*
		Cohort 1: CU (17)	MMSE	*
		Cohort 2: CU (36)	MMSE	*
Osborn et al. (2019)	NfL	Early MCI (27) MCI (132)	Episodic memory composite	*
			Executive function composite	*
			BNT	*
			ANT	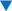
			WAIS‐IV coding	*
			DKEFS number sequencing	*
			Hooper visual organisation test	*
		CU (174)	Episodic memory composite	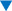
			Executive function composite	*
			BNT	*
			ANT	*
			WAIS‐IV coding	*
			DKEFS number sequencing	*
			Hooper visual organisation test	*
		Total (333)	Episodic memory composite	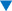
			Executive function composite	*
			BNT	*
			ANT	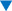
			WAIS‐IV coding	*
			DKEFS number sequencing	*
			Hooper visual organisation test	*
Portelius et al. (2015)	Ng	AD (95)	MMSE	*
			ADAS‐cog	*
		pMCI (105)	MMSE	*
			ADAS‐cog	*
		sMCI (68)	MMSE	*
			ADAS‐cog	*
		CU (110)	MMSE	*
			ADAS‐cog	*
Racine et al. (2016)	NfL	MCI + CU (70)	CPAL errors‐ visual memory	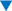
			GMCT moves/sec ‐speed of visual processing	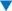
			GML errors	*
			GMR errors	*
			OCL accuracy	*
			ONB accuracy	*
			TWOB accuracy	*
			AVLT delayed	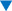
			Logical memory delayed	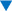
			BVMT‐R delayed	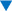
Rojas et al. (2018)	NfL	PSP (50)	RBANS	*
			Colour trails 1	*
			Colour trails 2	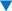
			Letter‐number sequencing	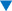
			Phonemic fluency	*
Rolstad, Berg, et al. (2015)	NfL	Dementia‐ vascular (65)	Attention composite	*
			Learning/memory composite	*
			Visuospatial composite	*
			Language composite	*
			Executive function composite	*
		Dementia‐ non‐vascular (128)	Attention composite	*
			Learning/memory composite	*
			Visuospatial composite	*
			Language composite	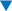
			Executive function composite	*
		MCI‐ vascular (86)	Attention composite	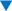
			Learning/memory composite	*
			Visuospatial composite	*
			Language composite	*
			Executive function composite	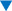
		MCI‐ non‐vascular (175)	Attention composite	*
			Learning/memory composite	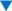
			Visuospatial composite	*
			Language composite	*
			Executive function composite	*
		SCI‐ vascular (48)	Attention composite	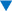
			Learning/memory composite	*
			Visuospatial composite	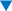
			Language composite	*
			Executive function composite	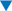
		SCI‐ non‐vascular (120)	Attention composite	*
			Learning/memory composite	*
			Visuospatial composite	*
			Language composite	*
			Executive function composite	*
Rolstad, Jakobsson, et al. (2015)	NfL	CU (71)	Memory composite	*
			Executive functions composite	*
			Visuospatial composite	*
			Attention composite	*
			Verbal functions composite	*
Sancesario et al. (2020)	Ng	CU (30)	MMSE	*
Sandelius et al. (2019)	GAP‐43	AD (275)	MMSE	*
		MCI (84)	MMSE	*
		CU (43)	MMSE	*
		FTD (39)	MMSE	*
		DLB (27)	MMSE	*
		lvPPA (10)	MMSE	*
		svPPA (15)	MMSE	*
		PSP (18)	MMSE	*
		CBS (19)	MMSE	*
		Total sample (662; CU, MCI, AD, ALS, FTD, PD, PD‐MCI, PD‐dementia, DLB, lvPPA, svPPA, PSP, CBS, PCA)	MMSE	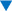
Sanfilippo et al. (2016)	Ng	AD (25)	MMSE	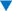
			CAMCOG	*
		MCI (50)	MMSE	*
			CAMCOG	*
		MCI‐AD (36)	MMSE	*
			CAMCOG	*
		CU (44)	MMSE	*
			CAMCOG	*
Santillo et al. (2019)	Ng	CU (20)	MCCB	*
Scherling et al. (2014)	NfL	Total: Asymptomatic FTD mutation carriers (8) bvFTD (45) nfvPPA (18) svPPA (16) CBS (17) AD (50) PSP (22) CU (47)	MMSE	*
			Rey‐Osterrieth figure	*
			DSF	*
			DSB	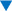
			TMT	*
			Stroop colour naming task	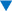
			BNT	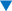
			ANT	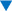
			CVLT	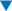
			Phonemic fluency	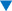
		bvFTD (45)	MMSE	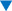
			Rey‐Osterrieth figure	*
			DSF	*
			DSB	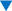
			TMT	*
			Stroop colour naming task	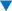
			BNT	*
			ANT	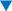
			CVLT	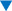
			Phonemic fluency	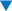
		nfvPPA (18)	MMSE	*
			Rey‐Osterrieth figure	*
			DSF	*
			DSB	*
			TMT	*
			Stroop colour naming task	*
			BNT	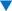
			ANT	*
			CVLT	*
			Phonemic fluency	*
Schindler et al. (2019)	Ng	Carriers of mutations in *PSEN1, PSEN2*, or *APP* (235)	DIAN cognitive composite	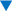
		Mutation non‐carriers (145)	DIAN cognitive composite	*
	SNAP‐25	Carriers of mutations in *PSEN1, PSEN2*, or *APP* (235)	DIAN cognitive composite	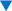
		Mutation non‐carriers (145)	DIAN cognitive composite	*
	VILIP‐1	Carriers of mutations in *PSEN1, PSEN2*, or *APP* (235)	DIAN cognitive composite	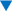
		Mutation non‐carriers (145)	DIAN cognitive composite	*
Sjogren et al. (2001)	NfL	Insignificant white matter changes (61; AD, SVD, CU)	MMSE	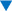
		Extensive white matter changes (14; AD, SVD, CU)	MMSE	*
Sjogren et al. (2000)	NfL	FTD (18)	MMSE	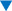
		AD (21)	MMSE	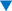
Skillback et al. (2014)	NfL	EAD (223)	MMSE	*
		AD (1194)	MMSE	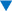
		FTD (146)	MMSE	*
		DLB (114)	MMSE	*
		VaD (465)	MMSE	*
		MIX (517)	MMSE	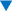
		PDD (45)	MMSE	*
		Dementia NOS (437)	MMSE	*
		Total (3103)	MMSE	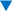
Sun et al. (2016)	Ng	ApoE ε4 carriers: AD (67) MCI (102) CU (27)	MMSE	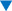
Swanson et al. (2016)	NPTX2	Total: AD (64) MCI (135) CU (86)	MMSE	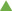
			ADAS‐Cog11	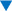
			Memory composite	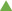
Teitsdottir et al. (2020)	NfL	AD CSF profile (28): SCI (2) MCI (9) AD (16) DLB (1)	Verbal episodic memory composite	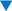
		Non‐AD CSF profile (14): SCI (10) MCI (13) DLB (1)	Verbal episodic memory composite	*
van der Ende et al., (2020)	NPTX2	Symptomatic genetic FTD (54)	MMSE	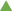
			TMT‐B	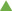
			Phonemic verbal fluency	*
		Presymptomatic genetic FTD (106)	MMSE	*
			TMT‐B	*
			Phonemic verbal fluency	*
van Steenoven et al. (2020)	NPTX2	DLB (85)	MMSE	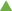
	NPTXR	DLB (85)	MMSE	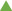
Wang (2019)	Ng	AD (81)	MMSE	*
		MCI (171)	MMSE	*
		CU (99)	MMSE	*
		Total (351)	MMSE	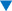
Wang, Zhou, and Zhang (2018)	SNAP‐25	AD (16) MCI (75) CU (55)	MMSE	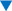
Wellington et al. (2016)	Ng	AD (100)	MMSE	*
		bvFTD (20)	MMSE	*
		svFTD (21)	MMSE	*
		DLB (13)	MMSE	*
		PSP (46)	MMSE	*
		CU (19)	MMSE	*
		Total (including PD, MSA, mood disorders)	MMSE	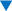
		AD‐like biomarker profile (151)	MMSE	*
		Non‐AD‐like biomarker (109)	MMSE	*
Xiao et al. (2017)	NPTX2	AD (30)	MMSE	*
			DSS	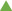
			BNT	*
			Phonemic verbal fluency	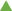
			Semantic verbal fluency	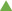
			WCST	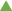
			Visual reproduction test	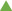
			Block design	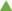
			CDT	*
			CVLT	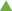
Zetterberg et al. (2016)	NfL	AD (95)	MMSE	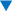
			ADAS‐cog	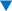
		pMCI (101)	MMSE	*
			ADAS‐cog	*
		sMCI (91)	MMSE	*
			ADAS‐cog	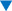
		CU (110)	MMSE	*
			ADAS‐cog	*
Zhang, Ng, et al. (2018)	VILIP‐1	Aβ + AD, MCI, CU (83)	MMSE	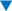
		Aβ‐ MCI, CU (38)	MMSE	*
Zhang, Therriault, et al. (2018)	SNAP‐25	AD (18)	MMSE	*
			ADAS‐cog	*
		sMCI (22)	MMSE	*
			ADAS‐cog	*
		pMCI (47)	MMSE	*
			ADAS‐cog	*
		CU (52)	MMSE	*
			ADAS‐cog	*

Abbreviations: ACE‐CZ, Addenbrookes Cognitive Examination‐Czech Version; AD, Alzheimers disease; ADAS‐Cog, Alzheimer Disease Assessment Scale‐Cognitive Subscale; ALS, amyotrophic lateral sclerosis; ANI, asymptomatic neurocognitive impairment; ANT, animal naming test; ApoE, apolipoprotein E; AVLT, Rey auditory verbal learning Test; Aβ‐, amyloid beta negative; Aβ+, amyloid beta positive; BMDB, brief mental deterioration battery; BNT, Boston naming test; BSRT, Buschke selective reminding test; bvFTD, behaviour variant FTD; BVMT‐R, Brief visuospatial memory test‐revised; BVMT, brief visuospatial memory test; CAMCOG, Cambridge cognitive examination; CBS, corticobasal syndrome; CDT, clock drawing test; CERAD, consortium to establish a registry for Alzheimers disease; CJD, Creutzfeldt‐Jacob disease; COWAT, controlled oral word association test; CPAL, continuous paired associate learning; CU, cognitive unimpaired; CVLT, California verbal learning test; DIAN, dominantly inherited Alzheimer network; DKEFS, Delis‐Kaplan executive function system; DLB, dementia with Lewy bodies; DSB, digit span backwards; DSF, digit span forwards; DSS, digit symbol substitution; EAD, early‐onset Alzheimers disease; FAB, frontal assessment battery; FTD, frontotemporal dementia; GMCT, groton maze times chase test; GML, groton maze learning test; GMR, groton maze learning test delayed recall; HAD, HIV‐associated dementia; lvPPA, logopenic variant primary progressive aphasia; MCCB, MATRICS consensus cognitive battery; MCI‐AD, mild cognitive impairment due to Alzheimers disease; MCI‐o, mild cognitive impairment not due to Alzheimers disease; MCI, mild cognitive impairment; MIX, mixed dementia; MMSE, mini mental state examination; MNCD, mild neurocognitive disorder; MND, motor neuron disease; MSA, multiple system atrophy; NfL, neurofilament‐light; nfvPPA, non‐fluent variant primary progressive aphasia; Ng, neurogranin; NOS, not otherwise specified; OCL, one‐card learning; ONB, one‐back memory; PASAT, paced auditory serial addition test; PCA, posterior cortical atrophy; PD, Parkinsons disease; PDD, Parkinsons disease dementia; pDLB, prodromal dementia with Lewy bodies; pMCI, progressive MCI; pNfH, phosphorylated neurofilament heavy; PSP, progressive supranuclear palsy; PVLT, Philadelphia verbal learning test; RBANS, repeatable battery for the assessment of neuropsychological status; SCWT, stroop colour word test; sMCI, stable MCI; SVD, small vessel disease; svPPA, semantic variant primary progressive aphasia; TMT‐B, trail making test B; TMT‐A, trail making test A; TWOB, two‐back memory; VaD, vascular dementia; WAIS, Wechsler adult intelligence scale; WCST, Wisconsin card sorting test; WE, Wernicke's encephalopathy.

A relationship between CSF NfL and neuropsychological performance was not consistently reported in MCI samples, although cognitive assessments used may have influenced findings. Three studies, with relatively large sample sizes, reported no significant association with MMSE scores (Bjerke et al., 2009; Bos et al., 2019; Zetterberg et al., 2016). However, several studies using other cognitive tests such as the ADAS‐Cog and cognitive composite scores reported associations with CSF NfL levels (Osborn et al., 2019; Rolstad, Berg, et al., 2015; Zetterberg et al., 2016). One study included participants with subjective cognitive impairment (SCI) and showed a significant association with a number of cognitive composite scores in those with a vascular burden (Rolstad, Berg, et al., 2015). Four studies pooled MCI and age‐matched CU samples and most reported a significant association with neuropsychological performance (Gifford et al., 2018; Osborn et al., 2019; Racine et al., 2016), while one reported no associations after controlling for demographics (Mielke, Syrjanen, Blennow, Zetterberg, Vemuri, et al., 2019). Five studies pooled AD, MCI and CU participants, and all reported a significant association with a number of neuropsychological assessments including the MMSE and ADAS‐Cog11(Bos et al., 2019; Dhiman et al., 2020; Mattsson et al., 2016; Mielke, Syrjanen, Blennow, Zetterberg, Skoog, et al., 2019; Teitsdottir et al., 2020). Interestingly, one study reported a stronger association in Aβ‐participants (Mattsson et al., 2016), while another reported a stronger association in Aβ + participants (Mielke, Syrjanen, Blennow, Zetterberg, Skoog, et al., 2019).

A significant association between CSF NfL and neuropsychological performance was consistently reported in 15 FTD studies; however, the cognitive assessment used may have influenced results. In FTD, three studies report significant moderate‐to‐weak relationships with MMSE scores (Alcolea et al., 2017; Delaby et al., 2020; Sjogren et al., 2000), although three studies showed no significant correlation (Abu‐Rumeileh et al., 2018; de Jong et al., 2007; Skillback et al., 2014). Despite a lack of association with MMSE scores, one study showed a weak but significant correlation with the frontal assessment battery (FAB)—a tool which is more sensitive to FTD (Dubois et al., 2000). Similarly, findings in studies of familial FTD were also mixed. One study found a significant correlation with MMSE scores in patients with a *C9orf72* mutation (Meeter, Gendron, et al., 2018), while another reported no association in those with mutations in the *MAPT, GRN*, or *C9orf72* genes (Meeter et al., 2016). Nevertheless, four studies examining subvariants of FTD consistently reported significant associations between CSF NfL and neuropsychological performance across subvariants (Meeter, Vijverberg, et al., 2018; Meeter et al., 2019; Rojas et al., 2018; Scherling et al., 2014).

Other types of dementia, including DLB and VaD, were investigated in five studies. Most studies reported no association between CSF NfL and MMSE in DLB (de Jong et al., 2007; Delaby et al., 2020; Skillback et al., 2014), although interestingly one showed a significant correlation in prodromal DLB. In two studies, NfL was correlated with neuropsychological performance in VaD and mixed dementia (Sjogren et al., 2001; Skillback et al., 2014). Finally, one study investigated HIV‐associated neurocognitive disorders (HAND) and while there were no associations between NfL and neuropsychological performance, there was a significant correlation with CSF levels of phosphorylated neurofilament heavy (pNfH) domains (McGuire et al., 2015).

Most studies including CU participants reported a significant association between CSF NfL and neuropsychological performance (Aschenbrenner et al., 2020; Gifford et al., 2018; Mielke, Syrjanen, Blennow, Zetterberg, Skoog, et al., 2019; Osborn et al., 2019); however, three reported no significant correlation (Bendlin et al., 2012; Bos et al., 2019; Hoglund et al., 2017). Moreover, studies using the MMSE consistently reported no significant correlation with CSF NfL levels, while most studies using other validated cognitive assessments reported significant results. One study included a younger sample (mean age = 37.8 years) and reported no significant association with cognitive composite test scores (Rolstad, Jakobsson, et al., 2015).

As reported in Table 4, CSF NfL appears to be related to neuropsychological performance in AD, MCI, CU and some forms of FTD. Conflicting results could be attributed to the cognitive assessment used; many studies employing the MMSE tended to report no associations, whereas more sensitive test scores appear to correlate with CSF NfL levels.

**TABLE 4:** Summary of results grouped by CSF marker and diagnosis

CSF marker	AD	MCI	CU	AD, MCI, CU Aβ+	AD, MCI, CU Aβ‐	AD, MCI, CU	MCI, CU	CUf	FTD‐related syndromes	DLB	VaD	EAD	PSP	CBS	Other
NfL	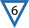	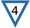	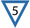	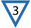	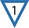	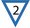	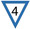		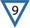	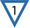	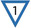		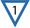	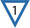	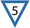
	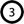	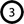	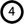		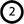		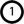	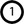	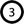	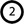	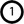	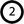	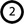	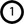	
Ng	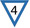	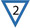		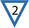	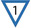	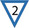	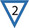	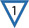							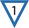
	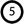	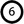	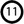	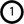	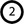		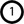		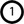	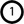			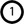		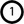
α‐Syn	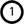		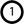												
β‐Syn	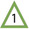														
Contactin‐2	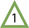														
GAP‐43	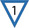 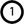	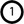	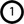						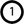	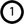			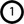	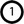	
NPTX1/2/R	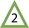 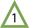			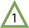	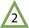	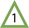			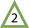	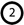					
Nrg1	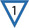	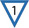													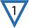
SNAP‐25	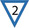			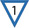		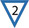									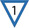
	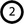	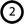	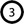		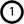										
Synaptotagmin‐1	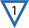 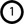	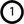	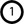												
		
VILIP‐1	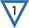			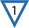											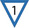
			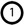		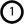										

*Note*: Blue inverted triangles (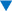) indicate a significant negative association, and green triangles (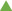), respectively, indicate a significant positive association between CSF marker levels and neuropsychological performance. Black circles indicate no significant associations. Numeric value within shape corresponds to number of studies with this finding.

Abbreviations: AD, Alzheimers disease; Aβ‐, amyloid beta negative; Aβ+, amyloid beta positive; CBS, corticobasal syndrome; CU, cognitively unimpaired; CUf, cognitively unimpaired with familial history of AD; DLB, dementia with Lewy bodies; EAD, early‐onset Alzheimers disease; FTD, frontotemporal dementia; GAP‐43, growth‐associated protein 43; MCI, mild cognitive impairment; NfL, neurofilament‐light; Ng, neurogranin; NPTX1/R, neuronal pentraxin 1/receptor; Nrg1, neuregulin‐1; PSP, progressive supranuclear palsy; SNAP‐25, synaptosomal‐associated protein 25; VaD, vascular dementia; VILIP‐1, visinin‐like protein 1; α‐syn, alpha‐synuclein; β‐syn, beta‐synuclein.

#### 3.3.2 Papers on CSF Ng

In total, 22 studies examined the association between CSF Ng and neuropsychological performance. Overall, CSF Ng was associated with neuropsychological performance in larger AD and MCI samples but not in CU or non‐AD dementias.

Nine studies examined the relationship with neuropsychological performance in AD samples. Some studies reported significant correlations with global cognition (Agnello et al., 2020; Bos et al., 2019; Jia et al., 2020; Sanfilippo et al., 2016), while a number of others reported no significant associations (De Vos et al., 2016; Hellwig et al., 2015; Portelius et al., 2015; Wang, 2019; Wellington et al., 2018). However, all studies reporting no association had sample sizes of fewer than 100 participants. In studies pooling AD, MCI and CU participants, sample sizes ranged from 193 to 770 and all three studies reported significant associations with neuropsychological performance (Bos et al., 2019; Galasko et al., 2019; Wang, 2019). In one study, this relationship was limited to Aβ+ participants (Bos et al., 2019), while in another it was independent of CSF Aβ and tau (Galasko et al., 2019). In carriers of autosomal dominant AD mutations in *PSEN1*, *PSEN2* or *APP* genes, one study reported a significant association between Ng and neuropsychological performance (Schindler et al., 2019). Finally, one study with a CU sample enriched for a familial history of AD and ApoE e4 carriers reported a weak correlation with neuropsychological performance (Casaletto et al., 2017).

Most studies examining MCI samples found no significant association between CSF Ng and MMSE or ADAS‐Cog scores (Bos et al., 2019; De Vos et al., 2016; Hellwig et al., 2015; Kvartsberg, Duits, et al., 2015; Portelius et al., 2015; Wang, 2019). Moreover, two studies using domain‐specific tests reported significant correlations (Headley et al., 2018; Mielke, Syrjanen, Blennow, Zetterberg, Skoog, et al., 2019). Interestingly, one study that reported no associations in MCI or SCI, however, did show a significant correlation between CSF Ng/BACE1 ratio and neuropsychological performance (Kirsebom et al., 2018).

Only two studies examined CSF Ng and neuropsychological performance in non‐AD dementias. Both showed no significant relationships in bvFTD, nfvPPA, PSP, DLB and non‐AD related MCI (Hellwig et al., 2015; Wellington et al., 2016).

Eleven studies reported no associations between CSF Ng and neuropsychological performance in CU samples (Bruno et al., 2020; Headley et al., 2018; Hoglund et al., 2017; Mielke, Syrjanen, Blennow, Zetterberg, Skoog, et al., 2019; Sancesario et al., 2020; Santillo et al., 2019; Schindler et al., 2019; Wang, 2019; Wellington et al., 2018). Cognitive domains assessed, immunoassay methods used or mean sample ages did not appear to influence results.

Overall, CSF Ng is associated with neuropsychological performance in AD studies (see Table 4) with large samples. Most studies reporting significant correlations had sample sizes of ~200 or above, while those reporting no relationship tended to have smaller samples. Findings for MCI were less convincing, as the majority of studies found no associations. No significant results were found for CU or non‐AD dementia samples.

#### 3.3.3 Papers on other CSF markers

Twenty‐two papers examined another CSF marker of interest. Overall, CSF NPTX2, and to a lesser extent CSF SNAP‐25, had the most promising evidence as markers associated with neuropsychological performance across diagnoses. Studies examining other CSF markers largely reported negative results.

A significant association between CSF NPTX2 and neuropsychological performance was consistently reported across studies. Three studies found a significant positive relationship with MMSE and domain‐specific assessments across the AD spectrum (Galasko et al., 2019; Swanson et al., 2016; Xiao et al., 2017), while one study reported no significant association (Boiten et al., 2021). Additionally, three studies reported associations in non‐AD dementias, namely, DLB (Boiten et al., 2021; van Steenoven et al., 2020) and FTD patients with *GRN, C9orf72* and *MAPT* mutations (van der Ende et al., 2020). Moreover, two studies found significant associations between MMSE scores and CSF NPTXR levels (Lim et al., 2019; van Steenoven et al., 2020). One study investigated CSF NPTX1 levels but reported no associations with MMSE scores (Begcevic et al., 2018).

Seven studies examined CSF SNAP‐25 across the AD‐spectrum, although findings were slightly more mixed. Two studies using the ultrasensitive SIMOA assay reported significant associations with neuropsychological performance in carriers of autosomal AD mutations and in a pooled sample of AD, MCI and CU, respectively (Galasko et al., 2019; Schindler et al., 2019). The use of other immunoassay methods did not appear to impact findings as studies using ELISAs and MS methods both reported significant (Brinkmalm et al., 2014; Jia et al., 2020; Wang, Zhou, & Zhang, 2018) and non‐significant (Öhrfelt et al., 2019; Zhang, Therriault, et al., 2018) associations. One papers did show an association between neuropsychological performance and a CSF SNAP‐25/Aβ^42^ ratio but not CSF SNAP‐25 alone.

Three studies reported significant correlations between MMSE scores and CSF VILIP‐1 in AD (Lee et al., 2008), a pooled sample of Aβ+ AD, MCI and CU participants (Zhang, Ng, et al., 2018) and in carriers of autosomal dominant AD mutations (Schindler et al., 2019). This relationship may be specific to those with Aβ pathology as one study reported no associations in a CU sample (Hoglund et al., 2017).

Few studies investigated the remaining CSF markers. Firstly, one small study showed a significant association between CSF nrg1 levels and MMSE scores in AD and MCI but not in non‐AD dementias (Mouton‐Liger et al., 2020). Secondly, CSF contactin‐2 levels were correlated with MMSE scores across the AD‐spectrum (Chatterjee, Del Campo, et al., 2018), but this failed to replicate in a validation cohort. Thirdly, CSF beta‐synuclein was correlated with MMSE scores but also failed to replicate in a validation cohort (Oeckl et al., 2020). No relationship was found between neuropsychological performance and alpha‐synuclein (Agnello et al., 2020; Bruno et al., 2020). Finally, findings concerning CSF GAP‐43 (Sandelius et al., 2019) and synaptotagmin‐1 were mixed; one small study reported significant associations with neuropsychological performance (Jia et al., 2020) while others failed to find such relationships (Öhrfelt et al., 2016; Sandelius et al., 2019).

Overall, CSF NPTX2 appears to be associated with neuropsychological performance across diagnoses (see Table 4). There was some evidence for an association with CSF SNAP‐25 across the AD‐spectrum; however, findings were somewhat mixed. Additionally, the few studies examining CSF VILIP‐1 levels reported significant relationships across the AD‐spectrum. Conversely, evidence for the remaining CSF markers is limited, owing to small samples and few studies examining such markers.

### 3.4 Heterogeneity

There was significant heterogeneity documented between the studies included in this review. Sources of variability were most evident in the number of difference cognitive assessments used. Although the MMSE was the most commonly employed test, many studies used cognitive composite scores, which hampered our ability to conduct a comparison between the studies. Moreover, across studies using the MMSE only, many non‐significant correlation coefficients were not reported.

Differences in statistical analyses also contributed to heterogeneity, while some studies used Spearman or Pearson correlations to analyse data and others used various regression models with different adjustment factors. For these reasons, a quantitative meta‐analysis of results was not possible.

## 4 DISCUSSION

We conducted a systematic review to investigate the relationship between CSF markers of synapse and neuronal loss and neuropsychological performance in dementia and typical ageing. Overall, the substantial heterogeneity between studies makes it difficult to draw firm conclusions on any markers associated with cognition. However, there may be evidence for an association between cognition and CSF NfL across dementia syndromes/cognitive ageing and CSF Ng in those with an AD‐like biomarker profile. There was some evidence CSF NPTX2 and SNAP‐25 are associated with cognition.

**FIGURE 2:**
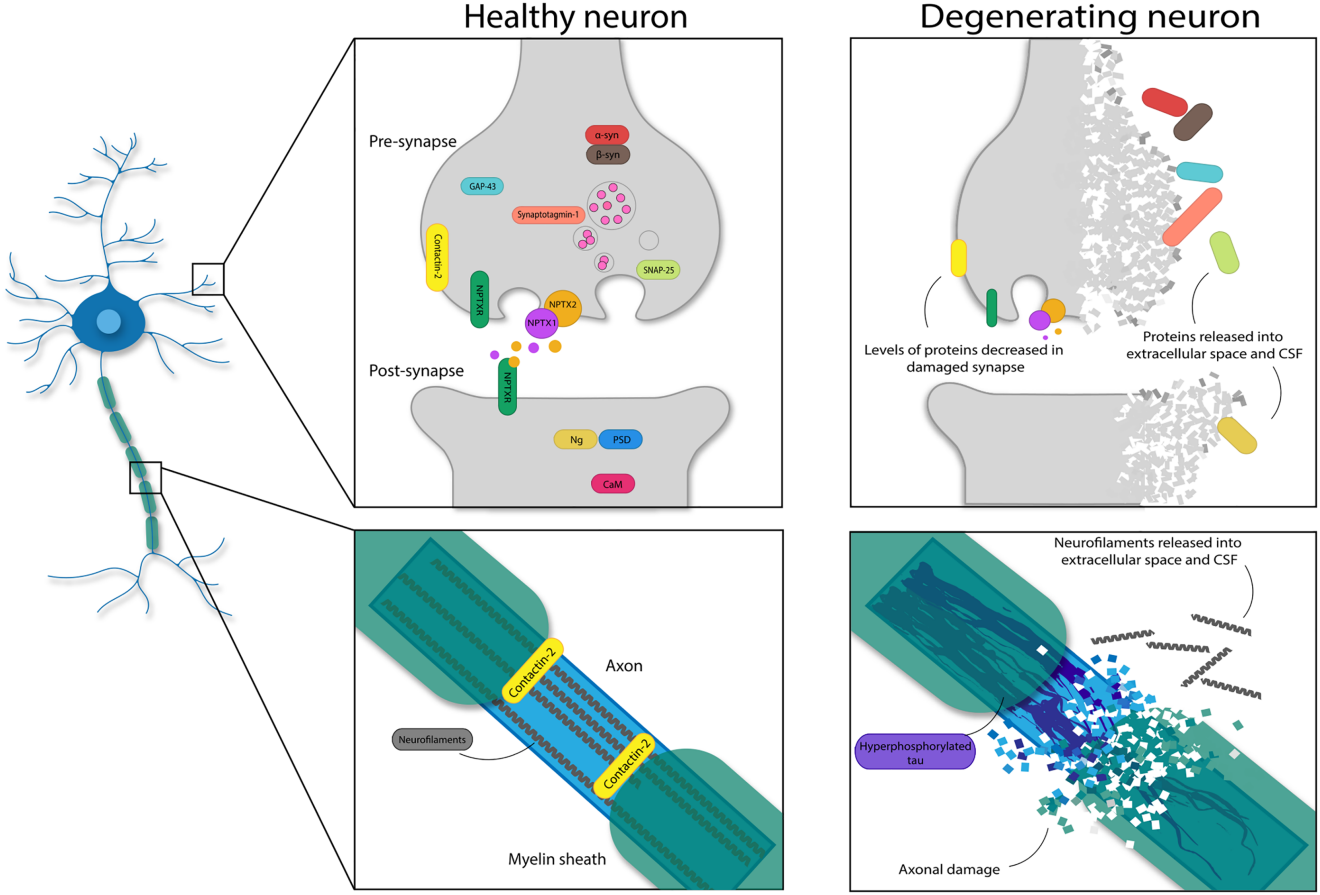
Schematic of localisation of synaptic and axonal markers included in the current review. Left: localisation in healthy synapses and axons. Right: possible mechanism of release into the cerebrospinal fluid (CSF) in degrading and damaged neurons

We found evidence for an association between CSF NfL and neuropsychological performance in AD, FTD and aged CU samples. There was some evidence for an association in MCI participants, but these findings were conflicting. Elevations of CSF NfL have been reported across neurodegenerative diseases and is thought to reflect global degeneration as neurofilaments ‘leak’ out of damaged axons into the CSF (see Figure 2). However, the lack of consistent findings for MCI samples was surprising. Most studies reporting non‐significant associations across diagnoses used the MMSE to assess cognitive impairment, while those using the ADAS‐Cog or domain‐specific tests tended to report significant correlations with CSF NfL levels. The MMSE is known to lack sensitivity, particularly in detecting MCI (Mitchell, 2009) and so it could be speculated that this test is not the most adequate to capture subtle cognitive impairments and therefore not a suitable tool for studies investigating potential biomarkers associated with cognition.

We also found some evidence that CSF Ng is associated with cognition in studies with large samples, possibly in Aβ+ participants (Bos et al., 2019) and Aβ+/Tau+ participants (Galasko et al., 2019). However, several studies focusing solely on participants with a clinical AD diagnosis reported no significant results. The use of the MMSE and small samples was a common feature of such studies indeed; those using larger samples tended to report significant associations. Meanwhile, CSF Ng was not associated with neuropsychological performance in non‐AD dementias. It is possible that Ng is specifically lost from synapses damaged by Aβ or tau, which are both associated with synaptotoxicity (Jackson et al., 2019; Koffie et al., 2009, 2012; Pickett et al., 2019) (see Figure 2). Indeed, CSF Ng was only associated with neuropsychological performance in a CU sample when enriched for a familial history of AD. However, the substantial heterogeneity between studies makes it difficult to draw firm conclusions on the use of CSF Ng as a biomarker associated with cognition.

The current review also highlighted other potential emerging biomarkers associated with cognition, namely, NPTX2 and SNAP‐25. CSF NPTX2 was consistently associated with neuropsychological performance in FTD, DLB and across the AD‐spectrum. In addition to its essential role at the synapse, low CSF NPTX2 levels are associated with hippocampal atrophy (Swanson et al., 2016), supporting its role as a biomarker of synapse dysfunction. Our findings suggest that CSF NPTX2 is not a disease‐specific marker of synapse loss but may instead reflect general synaptic dysfunction, although further research will be needed. CSF NPTX2, along with contactin‐2, was positively correlated with neuropsychological performance, unlike all other markers which had negative correlations. A potential explanation for these findings is that some synaptic and axonal proteins may leak out into the CSF after neuronal damage (those which show negative correlations with cognition); however, NPTXs and contactin‐2 levels may be reduced in surviving synapses, causing less to be secreted into the CSF as part of healthy synaptic turnover (see Figure 2).

SNAP‐25 was also a promising marker associated with cognition, although the evidence was less convincing and findings may have been influenced by small sample sizes. Prior to 2019, an ELISA assay available for CSF SNAP‐25 analysis was not available (Öhrfelt et al., 2019). With the growing accessibility of ELISA sampling technologies, we expect that further research will be able to employ larger sample sizes than those which are practical with mass‐spectrometry methods. Both studies using the ultrasensitive SIMOA immunoassay reported an association between SNAP‐25 levels and neuropsychological performance. Given the relatively low detected concentrations of CSF SNAP‐25 in the included studies, the improved sensitivity provided by SIMOA immunoassays may be more suited for future research.

While out of the scope of the current review, longitudinal studies of cognitive decline are also needed and useful. Cross‐sectional cognition can be dependent on several factors, such as age. While cross‐sectional age trends in cognitive measures have been reported to have a linear pattern, different samples with different ages may not be directly comparable (Salthouse, 2019). Longitudinal studies are needed to provide a direct measure of change with the same individuals assessed at each age. Longitudinal cohort studies such as the EPAD‐LCS (Ritchie et al., 2020; Solomon et al., 2018) may provide useful insights into how CSF markers relate to cognitive decline.

### 4.1 Beyond CSF markers

It is unlikely that a single CSF marker will act as a reliable biomarker for neuronal and synaptic changes affecting cognition. As assays become more sensitive and specific, a combination of CSF markers capturing different aspects of neurodegeneration may be a better correlate of cognition than single markers alone. However, CSF biomarkers are a relatively crude measure of brain function as regional differences cannot be examined. Incorporating both structural imaging (e.g. MRI) and functioning imaging (e.g. FDG‐PET and qEEG) along with cognitive testing is likely to provide a strong indication of neurodegeneration and cognitive status (Colom‐Cadena et al., 2020). Magnetic resonance imaging (MRI) can provide further information on neurodegeneration occurring in the brain. As one of the most widely used and accessible imaging methods, it is currently recommended in diagnostic criteria for AD (Jack et al., 2018). T1‐ and T2‐weighted images show different atrophy patterns and white matter alterations across different dementia syndromes (Harper et al., 2017), which all correlate with degree of the cognitive impairment (Bayram et al., 2018; Sudo et al., 2019; Wolk & Dickerson, 2011). The 7T MRI can provide further information about cognitive decline at an ultrahigh resolution, such as hippocampal subfield changes across dementias and MCI (McKiernan & OBrien, 2017).

Functional imaging can also provide information about brain functioning. Position emission tomography (PET) with 2 [(18)F]fluoro‐2‐deoxy‐D‐glucose (FDG‐PET) provides visualisation of the metabolic rate of glucose in the brain (Hoffman et al., 2000; Phelps et al., 1979) which is a direct index of synaptic functioning and an indirect index of synaptic density (Attwell & Iadecola, 2002; Rocher et al., 2003; Sokoloff, 1977). Reduced (18F) FDG uptake correlates with cognition in AD and MCI (Chiaravalloti et al., 2020; Landau et al., 2011). Recently, a direct measure of synapse density has been developed by targeting proteins critical for synaptic functioning (Finnema et al., 2016, 2018). PET ligands such as [^11^C]UCB‐J target synaptic vesicle glycoprotein 2A (SV2A), a ubiquitous protein expressed in pre‐synaptic terminals which is critical to synaptic function (Vogl et al., 2015). SV2A PET provides the opportunity to visualise synapses in vivo which is vital when investigating synapse loss. Decreased [^11^C]UCB‐J binding has been reported in early AD (Chen et al., 2018; Mecca et al., 2020) and correlates with episodic memory (Chen et al., 2018).

Additional functional imaging techniques, such as electroencephalography (EEG), provide a direct measure of neuronal field potentials. Reflecting the summed post‐synaptic potentials of excitatory and inhibitory neurons (Lopes da Silva, 2013), EEG is able to detect synapse dysfunction in vivo. Quantitative EEG analysis provides data reflecting neuronal circuit changes as a result of synapse dysfunction. Increases in delta (0.5–4 Hz) and theta (4–8 Hz) power bands, with a parallel decrease in alpha (8–13 Hz) and beta (13–30 Hz) power, have been reported in AD (Jelic et al., 2000). Furthermore, an increase in theta power is associated with clinical progression from SCI to MCI in those with Aβ pathology (Gouw et al., 2017), suggesting that changes in theta power may be associated with synapse dysfunction or loss. Magnetoencephalography (MEG) also records a signal based on post‐synaptic potentials; however, where EEG records electric potentials, MEG records the magnetic fields that are induced by electrical fields in the cortex (Lopes da Silva, 2013). Alterations have been reported in AD, MCI and SCI (López‐Sanz et al., 2018; Serrano et al., 2020; Xie et al., 2019), and increases in theta and beta2 power (20–30 Hz) have been reported in progressive MCI versus stable MCI (López et al., 2016). An increase in parietal delta power was found to increase the probability of conversion from MCI to AD by 350% (Fernández et al., 2006). Advantages of EEG and MEG include accessibility and non‐intrusive nature, as well as the excellent temporal resolution provided. Both of these functional techniques could contribute to an accurate readout of brain function at the network level.

With the exception of EEG and MRI in certain cases, the above methods are not part of routine practice. The costs associated with these methods, along with the invasive nature of CSF sampling and PET scans, could be a barrier to implementation in general practice. A biomarker detectable in the blood via a blood test would be more accessible, relatively invasive and most patients would be familiar with the procedure. A robust blood‐based biomarker of synapse loss or neuronal injury is not yet available; however, there is promising evidence for several markers.

Aβ and tau show promise as blood biomarkers for AD. Plasma Aβ is reduced in AD (Janelidze et al., 2016; Ovod et al., 2017; Zetterberg et al., 2011), correlates with CSF Aβ_42_ and can predict amyloid PET positivity (Nakamura et al., 2018). Plasma t‐tau and p‐tau levels are significantly increased in AD (Olsson et al., 2016; Randall et al., 2013; Zetterberg et al., 2013) and MCI (Yang et al., 2018). Plasma t‐tau correlates with cognitive decline in MCI ( Mielke et al., 2017), and plasma p‐tau181 is associated with both Aβ and tau PET (Mielke et al., 2018) and is more closely associated with AD neuropathology than a clinical diagnosis (Lantero Rodriguez et al., 2020). Blood levels of p‐tau217 are also elevated in AD and MCI and correlate with cognitive decline (Janelidze et al., 2020; Mattsson‐Carlgren et al., 2020). Blood levels of NfL show promise as a marker of general neurodegeneration; plasma or serum NfL levels are altered and correlate with MMSE scores in dementia syndromes and other neurodegenerative diseases (Al Shweiki et al., 2019; Khalil et al., 2020; Mattsson, Andreasson, et al., 2017; Sugarman et al., 2020; Zetterberg, 2016). However, not all CSF markers may be useful as blood biomarkers. In the CSF, Ng is a promising marker associated with cognition whereas in the blood, evidence suggests its use may be limited. While detectable in the blood, levels do not correlate with CSF Ng nor do they differ between AD and controls (De Vos et al., 2015; Kvartsberg, Portelius, et al., 2015). However, advancing technologies have made it possible to analyse neuron‐derived exosomes (NDEs) in blood which may offer increased sensitivity (Zetterberg, 2019). Indeed, a meta‐analysis reported a significant reduction of Ng plasma NDEs in AD and MCI (Liu et al., 2020). One study found an inverse correlation between GAP‐43, SNAP‐25, Ng and synaptotagmin‐1 NDEs and CSF levels of the protein, as well as a significant reduction in AD and MCI, and a significant correlation with MMSE scores (Jia et al., 2020). While this is promising evidence, the validation of blood biomarkers faces additional challenges. The CSF contains more neuronally derived molecules than blood (Zetterberg, 2019) which is particularly important to consider if the analyte of interest is expressed elsewhere in the body other than the brain, such as Ng expression in the lungs and kidneys which could explain the lack of correlation between blood and CSF levels (Díez‐Guerra, 2010). Blood biomarkers require sensitive and specific assays with meticulous validation studies (Zetterberg & Burnham, 2019), and the issues surrounding low reproducibility for CSF markers is also relevant for the validation of blood biomarkers.

### 4.2 Limitations

While this is the first known systematic review to examine CSF biomarkers associated with cognition in ageing and disease, it was not possible to conduct a meta‐analysis. An independent academic librarian was consulted with regard to the overall search strategy; however, they did not validate search terms. Furthermore, T.S.S. and D.A.G. were not blinded to studies when extracting data or rating the quality of studies which could introduce bias. Publication bias could also have affected the results of this review.

### 4.3 Recommendations

The current review reported conflicting findings between similar populations. While biologically important differences could explain these apparent discrepant findings, methodological heterogeneity could also be a contributing factor. We were unable to assess heterogeneity statistically; however, our review indicated substantial variability in methodology between studies. For example, differences in adjustment factors, cognitive tests and statistical analyses performed were some of the most common variations noted. A recent review has discussed low reproducibility as a common issue for biomarker findings (Mattsson‐Carlgren, Palmqvist, et al., 2020). The authors highlighted a number of sources of variability including cohort factors, assay factors, pre‐analytical factors and lack of validation methods. The field could improve on standardization with selecting a gold‐standard cognitive assessment, common adjustment factors, and the complete reporting of results. For novel biomarkers, validation cohorts are the most robust validation method (Mattsson‐Carlgren, Palmqvist, et al., 2020) and may improve the low reproducibility in the field. The overall quality of studies was good/fair. All studies clearly stated research objectives and most defined the study population clearly. However, only one of the included studies conducted a power analysis which limits confidence in findings, particularly in studies with smaller sample sizes.

To improve study quality and reporting, we recommend that future studies should address standardising cognitive assessments. The MMSE may not be the most appropriate tool due to floor and ceiling effects and a lack of sensitivity in detecting MCI (Mitchell, 2009). Other tests of global cognition such as The Repeatable Battery for the Assessment of Neuropsychological Status (Randolph, 1998) and the Addenbrooke's Cognitive Examination (Mathuranath et al., 2000) could be potential gold‐standard assessments for future studies, although further research is required. In addition to the assessment of global cognition, domain‐specific tests should also be used in future research. The International Working Group note a specific episodic memory disorder in AD which can be identified by tests that include list learning, such as the free and cued selective reminding test, paired associate learning and the Rey auditory verbal learning tasks (Dubois et al., 2014). Such tests are likely to be important in exploring potential biomarkers associated with disease‐specific cognitive impairments. A number of studies in the review used cognitive composite scores composed of various cognitive tools. These unstandardised composites contribute to variability in the field as they cannot be directly compared. Studies could improve on this by reporting the individual test scores in addition to composite scores or electing gold‐standard cognitive composites.

Future studies should also improve on the balanced reporting of data, as many studies did not report non‐significant correlation coefficients. Finally due to the nature of cohort studies, power analyses are unlikely to affect the final available sample but would still provide insight into whether individual studies are sufficiently powered to detect true relationships.

The reporting of sex and ethnicity differences was sparse. Concentrations of CSF biomarkers can vary with sex and ethnicity; CSF NfL is elevated in males, and elevations in CSF Ng have been reported for females (Mielke, 2020). Few studies have examined CSF marker changes across ethnicities; however, two studies report significant differences in CSF tau between African American and Caucasian groups (Garrett et al., 2019; Howell et al., 2017). Some studies in the current review controlled for sex (and less often for ethnicity), however, to work towards precision medicine, sex and ethnicity should be considered in the progression of cognitive decline, rather than treated as sources of random variability.

## 5 CONCLUSION

The current systematic review aimed to examine the relationship between CSF levels of markers for synaptic and neuronal damage with cognition in ageing and disease. Overall, heterogeneity between studies means no firm conclusions can be drawn from our results. We found some evidence for an association between neuropsychological performance and CSF NfL across diagnoses and CSF Ng in those with AD‐like pathology. Some studies found relationships with CSF NPTX2 across diagnoses. Recommendations for the field include the improvement of consistent analyses, measurements and reporting, as well as the exploration of important demographic differences in samples. In future research, a combination of CSF biomarkers of synaptic and neuronal loss and structural and functional imaging is likely to be a powerful tool for tracking changes affecting cognition and as a readout for interventions aiming to preserve cognitive function.

## CONFLICT OF INTEREST

The authors have no conflict of interest to report.

## AUTHOR CONTRIBUTIONS

TS, DK, TSJ, GM and CR conceived and designed the review. TS and DG performed the search, screened papers and extracted data. TSJ, DK, CR and GM provided supervision and guidance. TS wrote the original manuscript, and DG, TSJ, DK, GM and CR provided feedback and corrections.

### PEER REVIEW

The peer review history for this article is available at https://publons.com/publon/10.1111/ejn.15656.

## ABBREVIATIONS

α‐syn: Alpha‐synuclein
β‐syn: Beta‐synuclein
Aβ: Amyloid beta
ACE‐CZ: Addenbrooke's Cognitive Examination‐Czech Version
AD: Alzheimer's disease
ADAS‐Cog: Alzheimer's Disease Assessment Scale‐Cognitive Subscale
ADHD: Attention deficit hyperactivity disorder
ADNI: Alzheimer's Disease Neuroimaging Initiative
ALS: Amyotrophic lateral sclerosis
ANI: Asymptomatic neurocognitive impairment
ANT: Animal naming test
APOE: Apolipoprotein E
APP: Amyloid beta precursor protein
AVLT: Rey auditory verbal learning test
BACE: Beta‐secretase 1
BMDB: Brief mental deterioration battery
BNT: Boston naming test
BSRT: Buschke selective reminding test
bvFTD: Behavioural‐variant FTD
BVMT‐R: Brief Visuospatial memory test‐ revised
Ca^2+^
: Calcium
CaM: Calmodulin
CAMCOG: Cambridge cognitive examination
CBS: Corticobasal syndrome
CDR: Clinical dementia rating
CDT: Clock drawing test
CERAD: Consortium to establish a registry for Alzheimer's disease
CJD: Creutzfeldt‐Jakob disease (CJD)
COWAT: Controlled oral word association test
CNS: Central nervous system
CPAL: Continuous paired associate learning
CSF: Cerebrospinal fluid
CU: Cognitively unimpaired
CUf: Cognitive unimpaired with familial history of Alzheimer's disease
CV: Coefficient of variability
CVLT: California verbal learning test
DIAN: Dominantly inherited Alzheimer network
DKEFS: Delis‐Kaplan executive function system
DLB: Dementia with Lewy bodies
DSB: Digit span backwards
DSF: Digit span forwards
DSM‐III‐R: Diagnostic and statistical manual of mental disorders, 3rd edition revised
DSS: Digit symbol substitution
EAD: Early‐onset Alzheimer's disease
EEG: Electroencephalogram
ELISA: Enzyme‐linked immunosorbent assay
EMBASE: Excerpta Medica dataBASE
FAB: Frontal assessment battery
FDG: 2 [(18)F]fluoro‐2‐deoxy‐D‐glucose
FTD: Frontotemporal dementia
GAP‐43: Growth‐associated protein 43
GENFI: The Genetic Frontotemporal Initiative
GMCT: Groton maze times chase test
GML: Groton maze learning test
GMR: Groton maze learning test delayed recall
HAD: HIV‐associated dementia
HAND: HIV‐associated neurocognitive disorder
HIV: Human immunodeficiency virus
lvPPA: Logopenic variant primary progressive aphasia
IWG‐2: The International Working Group 2
LB: Lewy body
LTD: Long‐term depression
LTP: Long‐term potentiation
MAPT: Microtubule Associated Protein Tau
MCCB: MATRICS Consensus Cognitive Battery
MCI: Mild cognitive impairment
MCI‐AD: Mild cognitive impairment due to Alzheimer's disease
MCI‐o: Mild cognitive impairment not due to Alzheimer's disease
MEG: Magnetoencephalography
MIX: Mixed dementia
MMSE: Mini‐mental state examination
MNCD: Mild neurocognitive disorder
MND: Motor neuron disease
MRI: Magnetic resonance imaging
MS: Multiple sclerosis
MSA: Multiple system atrophy
NDE: Neuron‐derived exosomes
NfH: Neurofilament‐heavy
NfL: Neurofilament‐light
NfM: Neurofilament‐medium
nfvPPA: Nonfluent variant primary progressive aphasia
Ng: Neurogranin
NIA‐AA: National Institute on Ageing/Alzheimer's Association
NINCDS‐ADRDA: National Institute of Neurological and Communicative Disorders and the Alzheimer's Disease and Related Disorders Association
NOS: Not otherwise specified
NPTX: Neuronal pentraxin
NPTXR: Neuronal pentraxin receptor
Nrg1: Neuregulin‐1
OCL: One‐card learning
ONB: One‐back memory
P‐tau: Phosphorylated tau
PASAT: Paced auditory serial addition test
PCA: Posterior cortical atrophy
PD: Parkinson's disease
PDD: Parkinson's disease dementia
pDLB: Prodromal dementia with Lewy bodies
pMCI: Progressive MCI
pNfH: Phosphorylated neurofilament‐heavy
PET: Positron emission tomography
PPA: Primary progressive aphasia
PSEN: Presenilin
PSP: Progressive supranuclear palsy
PVLT: Philadelphia verbal learning test
RBANS: Repeatable Battery for the Assessment of Neuropsychological Status
SCI: Subjective cognitive impairment
SCWT: Stroop colour word test
sMCI: Stable mild cognitive impairment
SIMOA: Single molecule array
SNAP‐25: Synaptosomal‐associated protein 25
SNARE: Soluble NSF attachment protein receptor
SV2A: synaptic vesicle glycoprotein 2A
SVD: Small vessel disease
svPPA: Semantic variant primary progressive aphasia
T‐Tau: Total tau
TMT‐A: Trail making test A
TMT‐B: Trail making test B
TWOB: Two‐back memory
UCSD: University of California San Diego
VaD: Vascular dementia
VILIP‐1: Visinin‐like protein‐1
WAIS: Wechsler adult intelligence scale
WCST: Wisconsin card sorting test
WE: Wernicke's Encephalopathy

## Supporting information


**Supplementary Table S1**: Search terms
**Supplementary Table S2**: National Institute of Health Quality Assessment Tool for Observational Cohort and Cross‐Sectional Studies ratings for included studies

## Data Availability

No new data were generated in this systematic literature review.
