# Supplementary material for: Associations between cerebrospinal fluid markers and cognition in ageing and dementia: A systematic review
Source: Eur J Neurosci. 2022 May 12;56(9):5650–713. doi: 10.1111/ejn.15656 (PMC9790745; doi:10.1111/ejn.15656)
Supplement: Supplementary file 1 — Supplementary Table S1: Search terms Supplementary Table S2: National Institute of Health Quality Assessment Tool for Observational Cohort and Cross‐Sectional Studies ratings for included studies [file EJN-56-5650-s001.pdf]

Supplementary Table 1: Search terms

1	exp Dementia/ (159151)
2	dementia.tw. (99204)
3	dementia.mp. (118365)
4	exp Alzheimer Disease/ (90082)
5	Alzheimer's disease.mp. (111260)
6	Alzheimer's disease.tw. (108060)
7	Alzheimer.mp. (99838)
8	Alzheimer.tw. (25566)
9	early onset alzheimer.tw. (162)
10	late onset alzheimer.tw. (333)
11	early onset alzheimer.mp. (168)
12	late onset alzheimer.mp. (340)
13	alzheimer dementia.mp. (621)
14	alzheimer dementia.tw. (594)
15	dementia alzheimer.mp. (208)
16	dementia alzheimer.tw. (197)
17	alzheimer type dementia.mp. (713)
18	alzheimer type dementia.tw. (710)
19	dementia alzheimer type.mp. (51)
20	dementia alzheimer type.tw. (51)
21	exp Frontotemporal Dementia/ (2957)
22	fronto?temporal dementia.mp. (6629)
23	fronto?temporal dementia.tw. (5813)
24	(dementia adj5 lewy bod*).tw. (4649)
25	(dementia adj5 lewy bod*).mp. (4761)
26	dementia with lewy bodies.mp. (3786)
27	dementia with lewy bodies.tw. (3693)
28	exp Dementia, Vascular/ (6467)
29	vascular dementia.mp. (6197)
30	vascular dementia.tw. (5995)
31	dementia senile.mp. (42)
32	dementia senile.tw. (32)
33	senile dementia.mp. (2839)
34	senile dementia.tw. (2828)
35	senile dementia alzheimer type.mp. (27)
36	senile dementia alzheimer type.tw. (27)
37	Alzheimer type senile dementia.mp. (23)
38	alzheimer type senile dementia.tw. (23)
39	dementia primary senile degenerative.mp. (1)
40	disease alzheimer.mp. (129)
41	disease alzheimer.tw. (129)
42	alzheimer disease.mp. (95803)
43	alzheimer disease.tw. (15402)
44	familial alzheimer disease.mp. (309)
45	familial alzheimer disease.tw. (305)
46	sporadic alzheimer disease.mp. (155)
47	sporadic alzheimer disease.tw. (153)
48	exp Cognitive Dysfunction/ (14131)
49	mild cognitive impairment.mp. (15263)
50	cognitive dysfunction.mp. (25698)
51	cognitive dysfunction.tw. (12358)
52	mild cognitive impairment.tw. (14658)
53	cognitive impairment.mp. (52889)
54	cognitive impairment.tw. (51378)
55	cognitive decline.mp. (20005)
56	cognitive decline.tw. (19666)

57	exp Cognition/ (153665)
58	exp Memory/ (131781)
59	exp "Mental Status and Dementia Tests"/ (7153)
60	mini-mental state examination.mp. (12525)
61	mini-mental state examination.tw. (12420)
62	exp Neuropsychological Tests/ (172797)
63	RBANS.mp. (429)
64	RBANS.tw. (420)
65	exp Cognitive Aging/ (514)
66	cognitive aging.mp. (2371)
67	cognitive aging.tw. (1664)
68	cognitive ageing.mp. (287)
69	cognitive ageing.tw. (246)
70	age related cognitive decline.mp. (1152)
71	age related cognitive decline.tw. (1142)
72	neurodegenerati*.mp. (107535)
73	neurodegenerati*.tw. (99625)
74	exp Cerebrospinal Fluid/ (18386)
75	cerebrospinal fluid.mp. (113892)
76	cerebrospinal fluid.tw. (81799)
77	CSF.mp. (95526)
78	CSF.tw. (94530)
79	exp Neurofilament Proteins/ (5269)
80	neurofilament light chain.mp. (418)
81	neurofilament light chain.tw. (405)
82	neurofilament light polypeptide.mp. (48)
83	neurofilament light polypeptide.tw. (48)
84	NEFL.mp. (187)
85	NEFL.tw. (185)
86	NFL.mp. (1473)
87	NFL.tw. (1444)
88	neurofilament.mp. (10782)
89	neurofilament.tw. (8901)
90	neurofilament light.mp. (877)
91	neurofilament light.tw. (864)
92	light polypeptide.mp. (427)
93	light polypeptide.tw. (405)
94	exp Neurogranin/ (273)
95	neurogranin.mp. (426)
96	neurogranin.tw. (377)
97	RC3.mp. (190)
98	RC3.tw. (190)
99	p17.mp. (2213)
100	p17.tw. (2039)
101	BICKS.mp. (20)
102	BICKS.tw. (20)
103	protein kinase C substrate.mp. (356)
104	protein kinase C substrate.tw. (355)
105	NRGN.mp. (184)
106	NRGN.tw. (64)
107	exp Synaptosomal-Associated Protein 25/ (1614)
108	synaptosom* associated protein 25.mp. (1742)
109	synaptosom* associated protein 25.tw. (270)
110	exp GAP-43 Protein/ (2364)
111	GAP-43.mp. (3042)
112	GAP-43.tw. (2184)
113	growth associated protein 43.mp. (1198)
114	growth associated protein 43.tw. (1179)

115	protein F1.mp. (125)
116	protein F1.tw. (124)
117	neuromodulin.mp. (170)
118	neuromodulin.tw. (166)
119	neural phosphoprotein B-50.mp. (1)
120	exp Calmodulin/ (14092)
121	calmodulin.mp. (43103)
122	calmodulin.tw. (29083)
123	exp Calcium-Calmodulin-Dependent Protein Kinases/ (18328)
124	exp Calmodulin-Binding Proteins/ (5522)
125	calmodulin dependent protein kinase.mp. (8786)
126	calmodulin dependent protein kinase.tw. (5682)
127	calmodulin binding protein.mp. (541)
128	calmodulin binding protein.tw. (535)
129	(synap* protein adj5 cogniti*).mp. [mp=title, abstract, original title, name of substance word, subject heading word, floating sub-heading word, keyword heading word, organism supplementary concept word, protocol supplementary concept word, rare disease supplementary concept word, unique identifier, synonyms] (15)
130	(synap* protein adj5 cogniti*).tw. (15)
131	(synap* protein adj15 dementia).mp. (9)
132	(synap* protein adj15 dementia).tw. (9)
133	(synap* protein adj15 cerebrospinal fluid).mp. (6)
134	(synap* protein adj15 cerebrospinal fluid).tw. (6)
135	SNAP25.mp. (1378)
136	SNAP25.tw. (567)
137	synap* protein.mp. (1465)
138	synap* protein.tw. (1446)
139	synap* marker.mp. (290)
140	synap* marker.tw. (287)
141	or/1-73 (693628)
142	or/74-78 (165537)
143	or/79-140 (67285)
144	141 and 142 and 143 (470)

Supplementary Table 2: National Institute of Health Quality Assessment Tool for Observational Cohort and Cross-Sectional Studies ratings for included studies

Study	Was the research question or objective in this paper clearly stated?	Was the study population clearly specified and defined?	Was the participant rate of eligible persons at least 50%?	Were all the participants selected or recruited from the same or similar populations (including the same time period)? Were inclusion/exclusion criteria prespecified and applied uniformly?	Was a sample size justification, power description, or variance effect estimates provided?	For the analyses, were the exposure(s) of interest measured prior to the outcome(s) being measured?	Was the timeframe sufficient so that one could reasonably expect to see an association between exposure and outcome if it existed?	For exposures that can vary in amount or level, did the study examine different levels of the exposure as related to the outcome?	Were the exposure measures clearly defined, valid, reliable, and implemented consistently across all participants?	Was the exposure assessed more than once over time?	Were the outcome measures clearly defined, valid, reliable, and implemented consistently across all participants?	Were the outcome assessors blinded to the exposure status of participants?	Was loss to follow-up after baseline 20% or less?	Were key potential confounding variables measured and adjusted for statistically?	Quality Rating
Abu-Rumeileh et al. (2018)	Y	Y	NR	Y	N	N/A	N/A	Y	Y	N	Y	Y	N/A	Y	Good
Agnello et al. (2020)	Y	Y	NR	Y	N	N/A	N/A	Y	Y	N	Y	NR	N/A	N	Fair
Alcolea et al. (2017)	Y	Y	NR	N	N	N/A	N/A	Y	N	N	Y	NR	N/A	Y	Fair
Aschenbrenner 2020	Y	Y	NR	Y	N	Y	N/A	Y	Y	Y	Y	NR	N	Y	Good
Bartos et al. (2012)	Y	Y	NR	Y	N	N/A	N/A	Y	Y	N	Y	NR	N/A	NR	Fair
Begcevic et al. (2020)	Y	Y	NR	Y	N	N/A	N/A	Y	Y	N	Y	NR	N/A	N	Fair
Bendlin et al. (2012)	Y	Y	NR	Y	N	N/A	N/A	Y	Y	N	Y	NR	N/A	Y	Fair
Bjerke et al. (2009)	Y	Y	NR	N	N	N/A	N/A	Y	Y	N	Y	Y	N/A	Y	Good
Boiten et al. (2021)	Y	Y	NR	Y	N	CD	N/A	Y	Y	N	Y	CD	N/A	Y	Fair
Bos et al. (2019)	Y	Y	NR	Y	N	N/A	N/A	Y	Y	Y	Y	Y	N/A	Y	Good
Brinkmalm et al. (2014)	Y	Y	NR	N	N	N/A	N/A	Y	Y	N	Y	NR	N/A	NR	Fair
Bruno et al. (2020)	Y	N	NR	CD	N	N	N/A	Y	Y	N	Y	NR	N/A	N	Fair
Casaletto et al. (2017)	Y	Y	NR	Y	N	N/A	N/A	Y	Y	N	Y	Y	N/A	Y	Good
Chatterjee et al. (2018)	Y	Y	NR	Y	N	N/A	N/A	Y	Y	N	Y	Y	N/A	Y	Good
deJong et al. (2007)	Y	N	NR	CD	N	N/A	N/A	Y	Y	N	Y	NR	N/A	Y	Fair
Delaby et al. (2020)	Y	Y	NR	Y	N	N	N/A	Y	Y	N	Y	NR	N/A	Y	Fair
DeVos et al. (2016)	Y	N	NR	CD	N	N/A	N/A	Y	Y	Y	Y	NR	N/A	Y	Fair

Study	Was the research question or objective in this paper clearly stated?	Was the study population clearly specified and defined?	Was the participant rate of eligible persons at least 50%?	Were all the participants selected or recruited from the same or similar populations (including the same time period)? Were inclusion/exclusion criteria prespecified and applied uniformly?	Was a sample size justification, power description, or variance effect estimates provided?	For the analyses, were the exposure(s) of interest measured prior to the outcome(s) being measured?	Was the timeframe sufficient so that one could reasonably expect to see an association between exposure and outcome if it existed?	For exposures that can vary in amount or level, did the study examine different levels of the exposure as related to the outcome?	Were the exposure measures clearly defined, valid, reliable, and implemented consistently across all participants?	Was the exposure assessed more than once over time?	Were the outcome measures clearly defined, valid, reliable, and implemented consistently across all participants?	Were the outcome assessors blinded to the exposure status of participants?	Was loss to follow-up after baseline 20% or less?	Were key potential confounding variables measured and adjusted for statistically?	Quality Rating
Dhiman et al. (2020)	Y	Y	NR	Y	N	CD	N/A	Y	Y	N	Y	NR	N/A	Y	Fair
Galasko et al. (2019)	Y	Y	NR	CD	N	N/A	N/A	Y	Y	N	Y	Y	N/A	Y	Good
Gifford et al. (2018)	Y	Y	N	Y	N	N/A	N/A	Y	Y	N	Y	Y	N/A	Y	Good
Headley et al. (2018)	Y	Y	NR	Y	N	N/A	N/A	Y	Y	N	Y	NR	N/A	Y	Fair
Hellwig et al. (2015)	Y	Y	NR	Y	N	N/A	N/A	Y	Y	N	Y	Y	N/A	CD	Fair
Hoglund et al. (2015).	Y	Y	Y	Y	N	Y	N/A	Y	Y	N	Y	NR	N/A	Y	Fair
Jia et al. (2020)	Y	N	NR	N	N	N/A	N/A	Y	Y	N	Y	Y	N/A	CD	Fair
Kirsebom et al. (2018)	Y	Y	NR	N	N	N/A	N/A	Y	Y	Y	Y	NR	N A	Y	Fair
Kvartsberg et al. (2015)	Y	Y	NR	CD	N	N/A	N/A	Y	Y	Y	Y	Y	N/A	Y	Good
Lee et al. (2008)	Y	N	NR	CD	N	N/A	N/A	Y	Y	N	Y	NR	N/A	CD	Poor
Lim et al. (2019)	Y	Y	NR	Y	N	N/A	N/A	Y	Y	N	Y	CD	N/A	N	Fair
Mattsson et al. (2016)	Y	Y	NR	CD	N	N/A	N/A	Y	Y	Y	Y	NR	N/A	Y	Good
McGuire et al. (2015)	Y	Y	NR	Y	N	N/A	N/A	Y	Y	Y	Y	NR	N/A	N	Fair
Meeter et al. (2016)	Y	Y	NR	Y	N	N/A	N/A	Y	Y	N	Y	NR	N/A	Y	Fair
Meeter et al. (2018)	Y	Y	NR	Y	N	N/A	N/A	Y	Y	N	Y	Y	N/A	Y	Good
Meeter et al. (2019)	Y	Y	NR	N	N	N/A	N/A	Y	Y	Y	N	NR	N/A	Y	Fair
Meeter et al. (2017)	Y	N	NR	CD	N	N/A	N/A	Y	Y	N	Y	NR	N/A	Y	Fair
Mielke et al. (2019a)	Y	Y	NR	Y	N	N/A	N/A	Y	Y	N	N	NR	N/A	Y	Fair
Mielke et al. (2019b)	Y	Y	NR	Y	N	CD	N/A	Y	Y	N	Y	NR	N/A	Y	Good
Mouton-Liger et al. (2020)	Y	Y	NR	Y	N	CD	N/A	Y	Y	N	Y	Y	N/A	Y	Good
Oeckl et al. (2020)	Y	Y	NR	Y	N	N	N/A	Y	Y	N	N	Y	N/A	Y	Fair

Study	Was the research question or objective in this paper clearly stated?	Was the study population clearly specified and defined?	Was the participant rate of eligible persons at least 50%?	Were all the participants selected or recruited from the same or similar populations (including the same time period)? Were inclusion/exclusion criteria prespecified and applied uniformly?	Was a sample size justification, power description, or variance effect estimates provided?	For the analyses, were the exposure(s) of interest measured prior to the outcome(s) being measured?	Was the timeframe sufficient so that one could reasonably expect to see an association between exposure and outcome if it existed?	For exposures that can vary in amount or level, did the study examine different levels of the exposure as related to the outcome?	Were the exposure measures clearly defined, valid, reliable, and implemented consistently across all participants?	Was the exposure assessed more than once over time?	Were the outcome measures clearly defined, valid, reliable, and implemented consistently across all participants?	Were the outcome assessors blinded to the exposure status of participants?	Was loss to follow-up after baseline 20% or less?	Were key potential confounding variables measured and adjusted for statistically?	Quality Rating
Ohrfelt et al. (2016)	Y	Y	NR	Y	N	N/A	N/A	Y	Y	N	Y	CD	N/A	N	Fair
Ohrfelt et al. (2019)	Y	N	NR	CD	N	CD	N/A	Y	Y	CD	Y	CD	N/A	Yes	Fair
Osborn et al. (2019)	Y	Y	N/A	Y	N	N/A	N/A	Y	Y	N	Y	Y	N/A	Y	Good
Portelius et al. (2015)	Y	Y	NR	Y	N	N/A	N/A	Y	Y	N	Y	NR	N/A	Y	Fair
Racine et al. (2016)	Y	Y	NR	Y	N	N/A	N/A	Y	Y	N	Y	NR	N/A	Y	Fair
Rojas et al. (2018)	Y	Y	Y	Y	N	N/A	N/A	Y	Y	N	Y	NR	N/A	Y	Fair
Rolstad et al. (2015a)	Y	Y	NR	Y	N	N/A	N/A	Y	Y	N	Y	NR	N	Y	Fair
Rolstad et al. (2015b)	Y	Y	N	Y	N	N/A	N/A	Y	Y	N	Y	Y	N/A	Y	Good
Sancesario et al. (2020)	Y	Y	NR	Y	N	CD	N/A	Y	Y	N	Y	Y	N/A	Y	Good
Sandelius et al. (2019)	Y	N	NR	Y	N	N/A	N/A	Y	Y	N	Y	Y	N/A	CD	Fair
Sanfilippo et al. (2016)	Y	N	NR	Y	N	N/A	N/A	Y	Y	N	Y	Y	N/A	CD	Fair
Santillo et al. (2019)	N	N	NR	Y	N	N/A	N/A	Y	Y	N	Y	NR	N/A	Y	Poor
Scherling et al. (2014)	Y	N	NR	Y	N	N/A	N/A	Y	Y	N	Y	NR	N/A	CD	Poor
Schindler et al. (2019)	Y	Y	NR	Y	N	N/A	N/A	Y	Y	Y	Y	NR	N/A	Y	Fair
Sjogren et al. (2001)	Y	Y	NR	Y	N	N/A	N/A	Y	Y	N	Y	NR	N/A	Y	Fair
Sjogren et al. (2000)	Y	Y	NR	Y	N	N/A	N/A	Y	Y	N	Y	Y	N/A	Y	Fair
Skillback et al. (2014)	Y	Y	NR	Y	N	Y	N/A	Y	Y	N	Y	Y	N/A	Y	Good
Sun et al. (2016)	Y	N	NR	CD	N	N/A	N/A	Y	Y	N	Y	CD	N/A	Y	Fair
Swanson et al., (201)6	Y	N	NR	Y	N	N/A	N/A	Y	Y	N	Y	CD	N/A	Y	Fair
Teitsdottir et al. (2020)	Y	Y	NR	Y	N	N/A	N/A	Y	Y	N	Y	NR	N/A	Y	Fair
VanDerEnde et al. (2020)	Y	Y	NR	Y	Y	Y	N/A	Y	Y	N	Y	Y	N/A	Y	Good

Study	Was the research question or objective in this paper clearly stated?	Was the study population clearly specified and defined?	Was the participant rate of eligible persons at least 50%?	Were all the participants selected or recruited from the same or similar populations (including the same time period)? Were inclusion/exclusion criteria prespecified and applied uniformly?	Was a sample size justification, power description, or variance effect estimates provided?	For the analyses, were the exposure(s) of interest measured prior to the outcome(s) being measured?	Was the timeframe sufficient so that one could reasonably expect to see an association between exposure and outcome if it existed?	For exposures that can vary in amount or level, did the study examine different levels of the exposure as related to the outcome?	Were the exposure measures clearly defined, valid, reliable, and implemented consistently across all participants?	Was the exposure assessed more than once over time?	Were the outcome measures clearly defined, valid, reliable, and implemented consistently across all participants?	Were the outcome assessors blinded to the exposure status of participants?	Was loss to follow-up after baseline 20% or less?	Were key potential confounding variables measured and adjusted for statistically?	Quality Rating
Van Steenoven et al. (2020)	Y	Y	NR	Y	N	N	N/A	Y	Y	N	Y	Y	N/A	Y	Good
Wang et al. (2019)	Y	Y	NR	Y	N	N/A	N/A	Y	Y	N	Y	NR	N/A	Y	Fair
Wang et al. (2018)	Y	Y	NR	Y	N	N/A	N/A	Y	Y	N	Y	NR	N/A	N	Fair
Wellington et al. (2016)	Y	Y	NR	Y	N	N/A	N/A	Y	Y	N	Y	Y	N/A	N	Fair
Xiao et al. (2017)	Y	N	NR	Y	N	N/A	N/A	Y	Y	N	Y	Y	N/A	N	Fair
Zetterberg et al. (2016)	Y	Y	NR	Y	N	N/A	N/A	Y	Y	N	Y	Y	N/A	Y	Good
Zhang et al. (2018a)	Y	Y	NR	Y	N	N/A	N/A	Y	Y	N	Y	CD	N/A	Y	Fair
Zhang et al. (2018b)	Y	Y	NR	Y	N	N/A	N/A	Y	Y	N	Y	CD	N/A	Y	Fair
